# Lysyl Oxidases as Targets for Cancer Therapy and Diagnostic Imaging

**DOI:** 10.1002/cmdc.202300331

**Published:** 2023-09-04

**Authors:** Reik Löser, Manuela Kuchar, Robert Wodtke, Christin Neuber, Birgit Belter, Klaus Kopka, Lakshmi Santhanam, Jens Pietzsch

**Affiliations:** [a] Institute of Radiopharmaceutical Cancer Research, Helmholtz-Zentrum Dresden Rossendorf, Bautzner Landstraße 400, 01328 Dresden (Germany); [b] Faculty of Chemistry and Food Chemistry, School of Science, Technische Universität Dresden, Mommsenstraße 4, 01069 Dresden (Germany); [c] Departments of Anesthesiology and Critical Care Medicine and Biomedical, Engineering, Johns Hopkins University, Baltimore, MD 21287 (USA)

**Keywords:** enzyme inhibitors, extracellular matrix, posttranslational modification, quinoproteins, radiotracers

## Abstract

The understanding of the contribution of the tumour microenvironment to cancer progression and metastasis, in particular the interplay between tumour cells, fibroblasts and the extracellular matrix has grown tremendously over the last years. Lysyl oxidases are increasingly recognised as key players in this context, in addition to their function as drivers of fibrotic diseases. These insights have considerably stimulated drug discovery efforts towards lysyl oxidases as targets over the last decade. This review article summarises the biochemical and structural properties of theses enzymes. Their involvement in tumour progression and metastasis is highlighted from a biochemical point of view, taking into consideration both the extracellular and intracellular action of lysyl oxidases. More recently reported inhibitor compounds are discussed with an emphasis on their discovery, structure-activity relationships and the results of their biological characterisation. Molecular probes developed for imaging of lysyl oxidase activity are reviewed from the perspective of their detection principles, performance and biomedical applications.

## Graphical Abstract

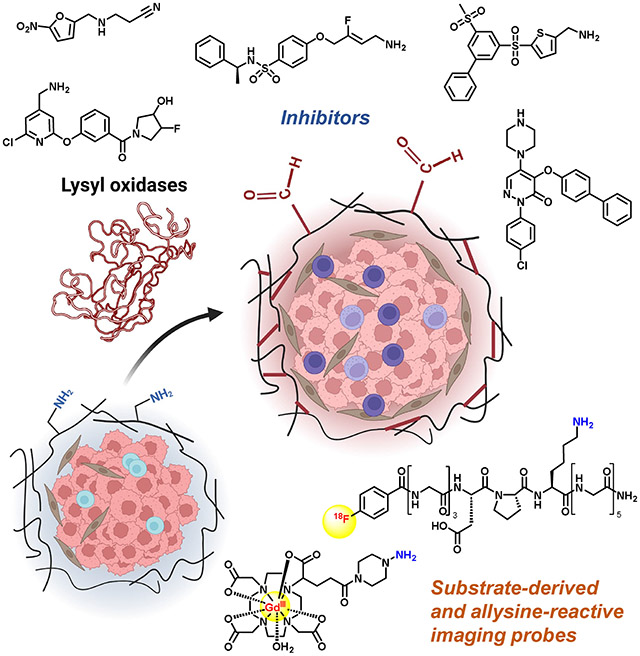

## 1. Introduction

The insights into the pathological significance of lysyl oxidases as catalysts of post-translational modification of protein-bound lysine residues for fibrotic and especially cancer diseases have grown considerably over the past two decades. Despite this potential, addressing this class of enzymes for therapeutic and diagnostic purposes has been exploited only to a limited extent. This is due, among other things, to difficulties in activity determination of these enzymes, and lack of structural knowledge. Nevertheless, the development of inhibitors and probes for addressing lysyl oxidases has experienced a significant upswing in the last 5 years.

In this review article, we intend to summarise biochemical and structural aspects of these unique enzymes, which is followed by an attempt to provide an overview on the molecular mechanisms for their function in tumour progression and metastasis. The function of lysyl oxidases in the pathogenesis of fibrotic disease is not reviewed in detail. Lysyl oxidases are furthermore involved in cardiovascular homeostasis and diseases, which is also beyond the scope of this review article but this function will be briefly highlighted at the end of section 3.3.

In the last two chapters recent trends in the development of inhibitors and molecular probes for detecting lysyl oxidases in biological specimens and for imaging at the organismic level will be highlighted and discussed. Selected material included in this article was taken from the habilitation thesis of the first author.^[1]^

## 2. Biochemical and Structural Aspects of Lysyl Oxidases

**Figure 1.**
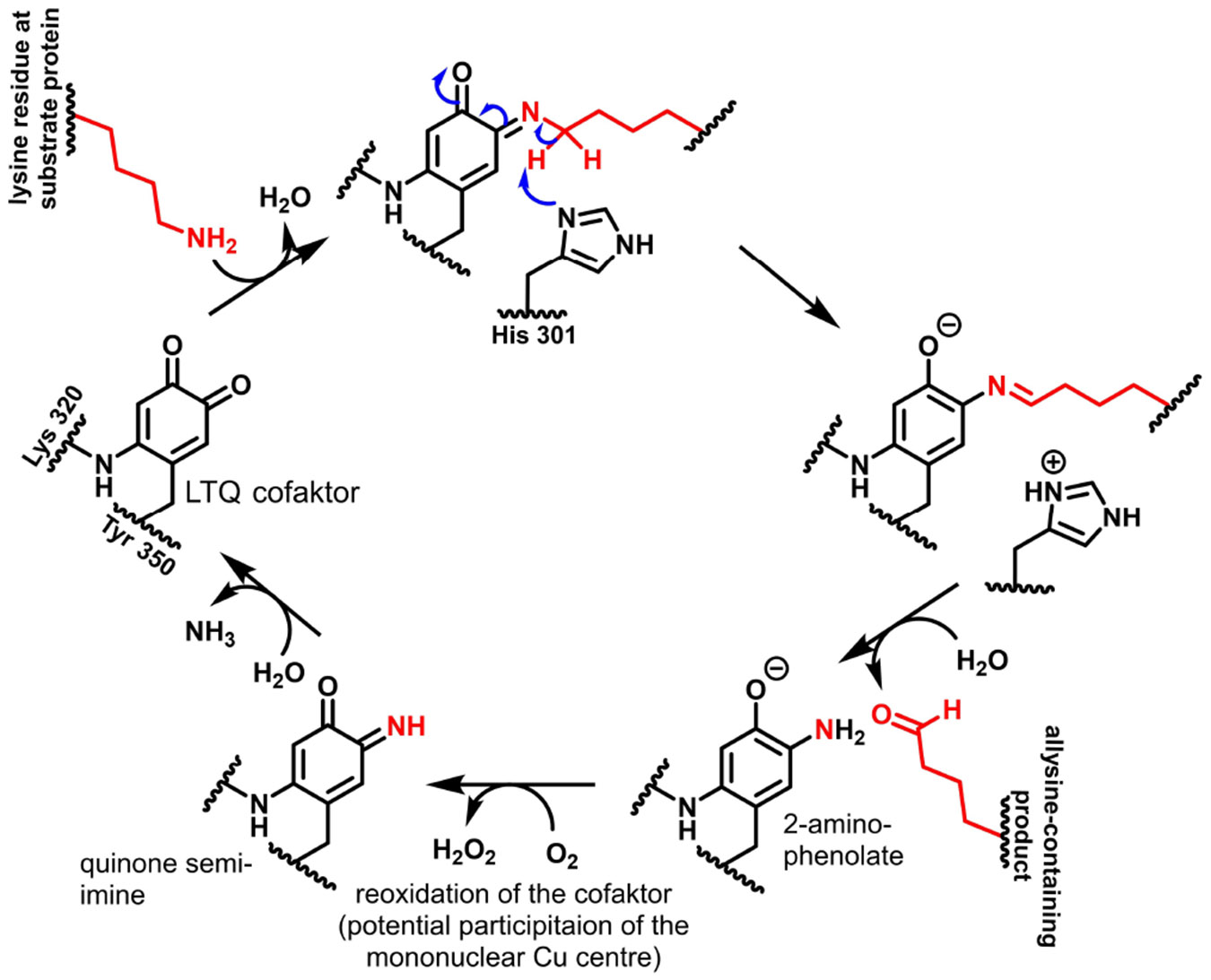
Mechanism of catalysis of lysyl oxidases. Numbering of the amino acid residues is based on the human LOX isozyme. For clarity, the mononuclear copper centre is omitted in the figure.

Lysyl oxidases catalyse the oxidative deamination of protein-bound lysine residues, converting them into allysine. In this process, oxygen, which is converted to hydrogen peroxide during the enzyme-catalysed reaction, serves as an electronaccepting cosubstrate. The nitrogen of the ε-amino group is hydrolytically released as ammonia (Figure 1). Such enzymatic activity was first demonstrated and described in 1968 at the National Institutes of Health (Bethesda, USA) by Sheldon R. Pinnell and George R. Martin in bone tissue of chicken embryos.^[2]^ In humans, a total of five proteins with lysyl oxidase activity are known. In addition to the prototypical lysyl oxidase (LOX), these are the so-called LOX-like isozymes LOXL1-4, which are encoded by gene segments on chromosomes 5, 15, 8, 2, and 10, respectively.^[3]^ The occurrence of lysyl oxidases is not restricted to vertebrates; rather, these enzymes appear to be distributed across all metazoans. Their evolutionary origin is apparently closely linked to the development of the extracellular matrix (ECM).^[4]^ With regard to the pathophysiological implications of lysyl oxidase-catalysed reactions, in addition to the transformation of the nucleophilic functionality of the primary amine to the highly electrophilic carbonyl group, the concomitant change in charge state is significant for the modification of the physicochemical properties of the substrate proteins. The physiological influence of the potentially cytotoxic products ammonia and hydrogen peroxide, which are released stoichiometrically in the course of the enzymatic reaction, should not be underestimated.

Enzymes that convert the kinetically inert triplet oxygen^[5]^ generally do so with the help of transition metal ions and/or prosthetic organic groups which can mediate one-electron transitions.^[6]^ Therefore, in order to elucidate the catalytic mechanism, it was obvious to search for such cofactors in lysyl oxidase as well. Initially, a copper ion associated with lysyl oxidase was identified^[7]^ and pyridoxal phosphate was mistakenly assumed to be the organic cofactor.^[8]^ Kagan and coworkers succeeded in identifying the structure of the cofactor as lysyl-tyrosyl-quinone (LTQ)^[9]^ and thus discovered a novel enzyme-associated prosthetic group that appears to exist only in lysyl oxidases.^[10]^ However, it appears worth mentioning that LTQ and indophenols derived from it have also been found as a cross-linking elements in ranasmurfin, a protein from the foam nests of the tropical frog *Polypedates leucomystax.*^[11]^ This covalently bound cofactor is formed by the residues Tyr 355 and Lys 320 (numbering for human LOX), where first the tyrosine residue is oxidised in the presence of oxygen mediated by the mononuclear copper centre to dopaquinone, which reacts with the ε-amino group of Lys 320 under intrachain bridging to the 4-aminocatechol, whose oxidation by oxygen leads to the quinone form of LTQ.^[9,12]^ The LTQ cofactor of lysyl oxidases is thus structurally, functionally and biogenetically closely related to the topaquinone cofactor (TPQ; 2,4,5-trihydroxyphenylalanine-derived ortho-benzoquinone) found in plasma amine oxidase and other copper-dependent amine oxidases.^[9]^ Thus, by knowing LTQ as a cofactor, a plausible mechanism for the lysyl oxidase-catalysed oxidation of primary amines can be formulated on the basis of kinetic studies,^[13]^ which is supported by results of studies on model compounds.^[10,14]^ In this process, condensation initially occurs between one of the carbonyl groups (the one in the para position of the amino group of Lys 320) with the ε-amino group of the lysine residue in the substrate. Mediated by deprotonation of the ε-methylene group, with His 303 acting as a general base,^[15]^ this quinone semi-imine tautomerises to aldimine and subsequent hydrolytic release of the allysine product leads to a 2-aminophenolate intermediate, which is oxidised by oxygen to an unsubstituted quinone semi-imine under the formation of hydrogen peroxide. The hydrolytic release of ammonia from this second quinone imine intermediate regenerates the LTQ cofactor for another catalytic cycle (Figure 1).

**Figure 2.**
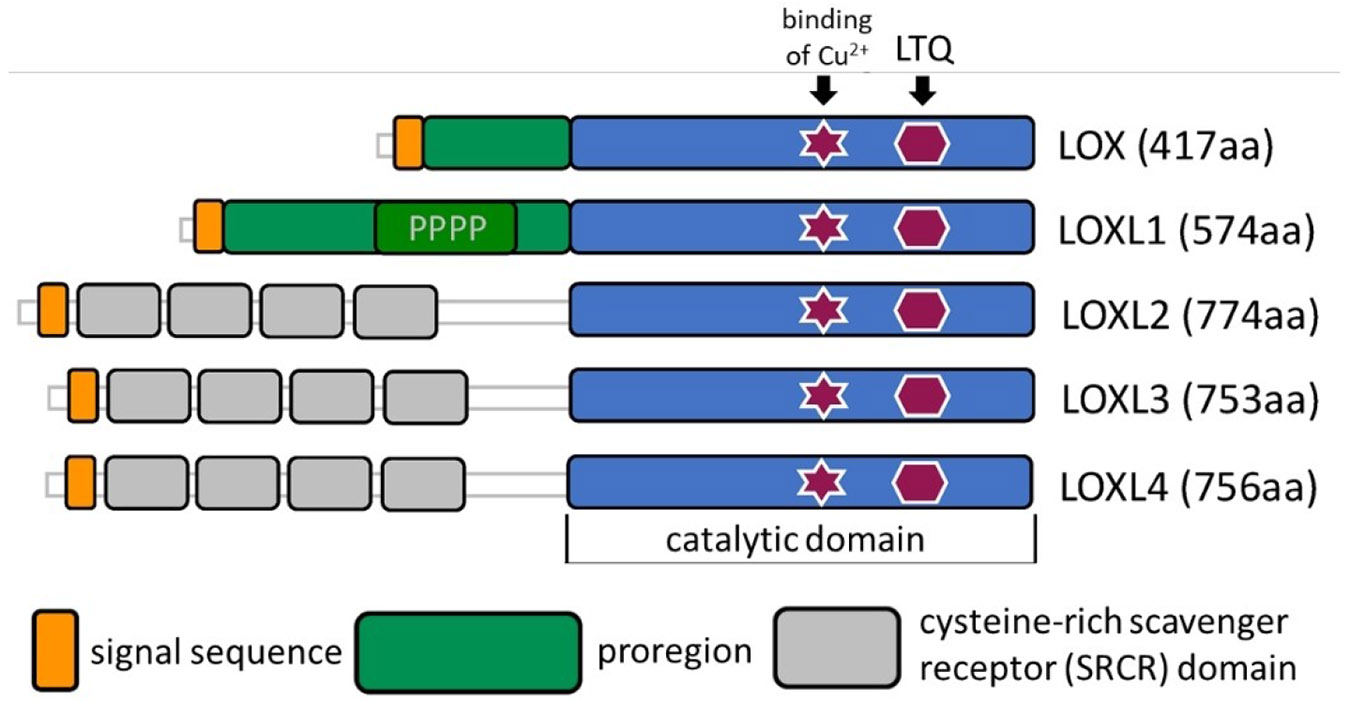
Domain structure of the lysyl oxidase isozymes. PPPP = proline-rich domain. Taken with permission from Amendola *et al.*^[30]^ and edited.

The bovine lysyl oxidase isolated from aortic tissue was known to have a molecular weight of 32 kDa,^[16]^ whereas a molecular mass of 46 kDa was deduced from the analysis of the corresponding gene segment.^[17]^ The difference is due to the presence of an N-terminal propeptide, which is removed after secretion of the proenzyme by the metalloprotease procollagen C-peptidase (bone morphogenetic protein-1, BMP-1). Numerous extracellular proteins have been identified as interaction partners for the isolated LOX propeptide, including various glycosaminoglycans, matrix metalloprotease (MMP)-2 and transglutaminase 2.^[18]^ The C-terminal, 204 amino acid catalytic domain of the prototypical LOX is also present in all other human lysyl oxidase isozymes, whereas a propeptide is otherwise only found in LOXL1. LOXL1 additionally has a proline-rich domain N-terminally upstream of the catalytic domain. In contrast, the LOX-like 2–4 isozymes have neither a propeptide nor the proline-rich domain. These three lysyl oxidase isozymes share the presence of four cysteine-rich scavenger receptor (SRCR) domains N-terminal to the oxidase region. The function of these domains is still largely unknown, although it is thought that they mediate interaction with fibrillar collagens as cross-linking substrates.^[4b]^ As proteins that are secreted extracellularly via the classical pathway, passing through the endoplasmic reticulum and Golgi apparatus, all lysyl oxidases have a 21–25 amino acid N-terminal signal peptide (Figure 2). The C-terminal catalytic domains of LOXL1-4 have sequence identities and homologies to LOX of at least 49 and 67%, respectively (Table 1). In addition to the domain responsible for the formation of the LTQ cofactor, histidines 289, 292, 294, 296 and 303 associated with the copper-binding centre are conserved in all five isoforms, with only histidines 292, 294 and 296 being involved in the coordinative binding of the Cu^2+^ ion.^[19]^ One ring N atom of each of the histidines mentioned and one water molecule form the equatorial coordination sphere of the Cu^2+^ ion. Carbonyl O atoms of amino acid residues that have not been identified with certainty presumably act as further axial ligands, resulting in the stretched-octahedral coordination geometry typical of Cu^2+^.^[7b,20]^ The association of copper with the enzyme occurs in the Golgi apparatus and is mediated by the copper transporter ATP7 A.^[21]^ The function of the Cu^2+^ ion is apparently limited to the oxidative formation of the LTQ factor; it is presumably not involved in the catalysis itself (Figure 1).^[22]^ In addition, ten conserved cysteine residues were identified in the catalytic domains, which form five disulfide bridges,^[23]^ whereby the connectivities vary between the individual isoforms.^[24]^ A segment of approximately 70 amino acid residues at the C-terminus (Glu 345-Pro 416), which partially overlaps with the catalytic domain (LTQ-forming sequence), shows similarity to cytokine receptor domains in all human lysyl oxidases. The function of this domain, similar to that of the SRCR domains, remains largely unexplored.

For the isolation and purification of lysyl oxidase from various tissues such as the aorta, lung and placenta, its exceptional solubility and stability in the presence of urea (4-6 M) has been exploited.^[25]^ Retrospectively, the stability to this chaotropic agent is due to the stabilisation of the tertiary structure of the catalytic domain by the six intrachain bridges (five disulfide bonds and LTQ) mentioned above. Enzyme activity appears to be optimal in the presence of 1.2 M urea.^[26]^ In the absence of urea, LOX tends to aggregate, which is due to the formation of insoluble oligomers, presumably induced by the tyrosine-rich N-terminal sequence segment.^[27]^ The tendency of lysyl oxidase to aggregate has meant that it has so far resisted crystallisation attempts as a prerequisite for determining its three-dimensional structure by X-ray diffraction.^[27]^ Nevertheless, a breakthrough in structural elucidation has recently been achieved with the solution of the X-ray crystal structure of a human LOXL2 truncated around the two N-terminal SRCR domains at 2.4 Å resolution. However, the Cu^2+^ ion of the catalytic domain in this structure is replaced by a redox-inactive Zn^2+^ ion, which means that the LTQ cofactor is not formed. In the crystal structure, a Ca^2+^ ion was also detected in the catalytic domain, which is coordinatively bound by the side chains of Glu 722, Asn 727, Asn 728, Asp 549, and the backbone carbonyls of Leu 550 and Asp 724.^[28]^ These residues are also conserved in all other lysyl oxidase isozymes, but this calcium binding site remained previously undiscovered.^[29]^

**Figure 3.**
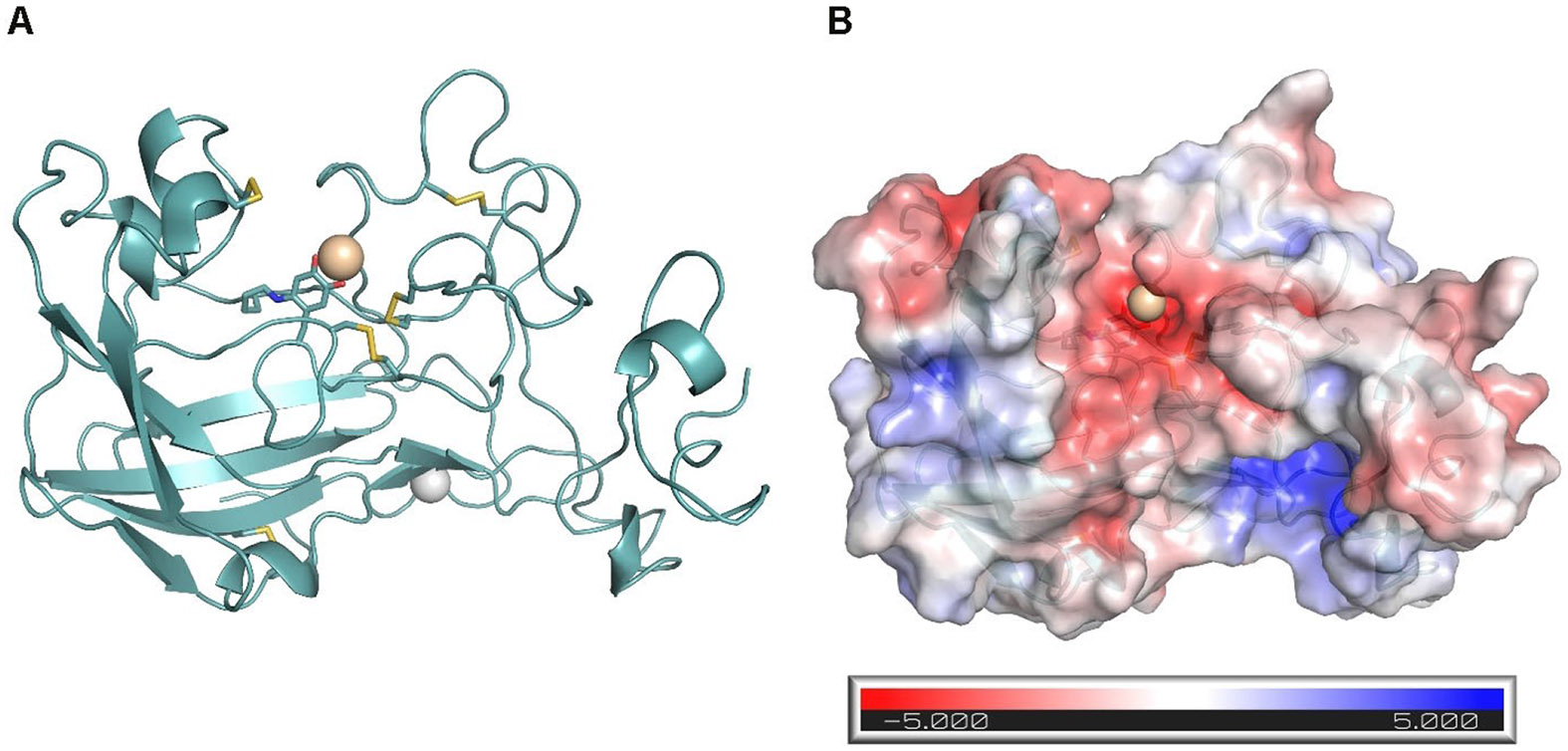
Structural model of human LOX according to Vallet *et al.*^[24a]^ A) Ribbon representation, B) Surface representation with charge scale [contour colour gradients: −5.0 kT/e (red) and + 5.0 kT/e (blue)]. Created with PyMOL from data published in.^[24a]^ The Cu^2+^ and Ca^2+^ ions are shown in ochre and grey, respectively.

Based on the crystal structure of the Zn^2+^-bound inactive catalytic domain of LOXL2, Vallet *et al.* have developed a homology model of Cu^2+^-containing LOX in which the LTQ cofactor is functionally formed.^[24a]^ This model further accounts for all experimental findings on the structure of the enzyme and its stability was validated by molecular dynamics simulations. The structure derived in this modelling study is shown in Figure 3. Previously, LOX structural models of lower quality have been published.^[27,31]^

Furthermore, on the basis of the mentioned crystal structure, the structure of the mature enzyme was recently modelled by Meier *et al.*, which has revealed that the major difference in the polypeptide backbone is restricted to a pentapeptide sequence (H652-K653-A654-S655-F656) around the lysine residue 653 forming the LTQ cofactor, which seems to change conformation from a β-sheet into a flexible loop prior to cross-linking between Lys 653 and the Tyr 689-derived dopaquinone.^[32]^ This finding is complemented by UV/Vis and Raman spectroscopic studies on 2-hydrazinopyridyl-inhibited LOXL2 and derived model compounds, which has revealed that the resulting pyridylazo-LTQ adduct acts as a tridentate ligand toward the Cu^2+^ ion.^[33]^ Taken together, the results of the modelling and spectroscopic studies suggest that the LTQ cofactor and the Cu^2+^ ion are in close proximity (< 3 Å) and solvent exposed, which is in stark contrast to the related Cu^2+^-dependent TPQ-containing amine oxidases, whose quinone cofacator is more buried, in line with their specificity for substrates of lower molar masses.^[32-33]^ Although a large number of low-molecular-weight amines are accepted as substrates by the lysyl oxidases,^[34]^ they presumably develop stronger catalytic activity towards protein-bound lysine residues.

A model of the full-length LOXL2 was derived on the basis of small-angle X-ray scattering (SAXS) and electron microscopy. These investigations have revealed that the enzyme exhibits a rod-like structure, consisting of a stalk, which comprises the four SRCR domains, like pearls on a string, followed by the catalytic domain at the tip.^[35]^

The recognition of lysine-containing proteins as substrates of lysyl oxidases seems to be influenced in a complex manner.^[19b,36]^ The requirements for the primary structure of peptide substrates were investigated in particular for substrates derived from collagen telopeptides.^[36]^ Furthermore, in the case of LOX, the turnover rates could be correlated with the net charges of the substrate proteins, since a large number of basic proteins with isoelectric points > 8 are readily oxidised by this enzyme, whereas proteins whose isoelectric points are lower are almost not converted by LOX.^[27,37]^ This finding is consistent with the structural model shown in Figure 3B, which indicates that the substrate-binding groove centred on the LTQ cofactor is characterised by a large-scale negative charge caused by numerous aspartate and glutamate residues near the cofactor. The isoelectric point of the mature LOX enzyme is 5.84 whereas that of the propeptide is 11.86. Thus, the binding of the latter to the active site of the catalytic domain should be electrostatically favoured.^[37c]^

Access and conversion of the extracellular scleroproteins elastin and collagen is regulated by a complex interplay of substrate and adaptor proteins and localised LOX activation. For the alanine-rich basic tropoelastin, it has been postulated that the interaction between this substrate and the LOX proregion is mediated by fibulin-5 and in the ternary complex the cleavage of the propeptide is catalysed by BMP-1.^[38]^ A similar function is attributed to fibulin-4.^[39]^ For type I collagen, oxidation of the lysine residue of the N-telopeptide in the triple-helical binary complex is electrostatically promoted.^[40]^ Furthermore, the cross-linking of type I and type II collagen seems to be supported by the leucine-rich proteoglycan fibromodulin.^[29,38a]^ It should be emphasised that the cross-linking reaction by imine (Schiff’s base) formation between an allysine and unmodified lysine residue or aldol condensation between two allysines, leading to dehydrolysinonorleucine and allysine aldol, respectively does not – as far as known – require enzymatic catalysis. Rather, these reactions seem to be supported by the spatial proximity of the reacting moieties, which also determined the conversion of the lysine residue in the previous step. In the case of collagen, the mentioned condensation reaction lead to “immature” cross-links, which can undergo further structural complex modifications to “mature” cross-links, as reviewed elsewhere.^[41]^ Very impressive cross-linking chemistry occurs in the case of tropoelastin, whose monomers of 49–69 kDa released from the cell assemble into clusters of premature elastin driven by liquid-liquid phase separation of the hydrophobic domains, which positions 4 lysine residues within hydrophilic α-helical KA domains in close proximity (reviewed in [42]). Three of these four lysine residues undergo lysyl-oxidase catalysed oxidation to allysine. These 3 allysine residues and the unmodified lysine formally undergo a Hantzsch-type cyclocondensation to dihydrodesmosine (3,4,5-substitution pattern) and dihydroisodesmosine (2,3,5-substitution pattern), which subsequently undergo oxidation to the corresponding pyridinium ions desmosine and isodesmosine, for which dehydrolysinonorleucine is proposed to act as an electron acceptor.^[43]^ The desmosine tetravalent cross-link is thought to arise from cyclocondensation between bivalent dehydrolysinonorleucine and the allysine-aldol cross-link, while in the case of isodesmosine an allysine aldol bivalent cross-link sequentially condenses with lysine (via formation of dehydromerodesmosine as trivalent cross-link) and subsequently allysine.^[42d,44]^ Both dehydrolysinonorleucine and allysine can occur as intra and intradomain cross-links, the latter both intra- and intermolecularly.^[44b,45]^

Although lysyl oxidase-initiated cross-linking seems to be common in extracellular scleroproteins, transformation of protein-bound lysine into allysine residues, which do not undergo further reaction, is considered as a distinct posttranslational modification.^[46]^

As will be explained in the next section, intracellular proteins can also be oxidatively modified by lysyl oxidases. This requires internalisation of the catalytically active extracellular enzymes, which has been experimentally confirmed for LOX.^[47]^ However, the mechanism underlying this process has not yet been clarified.

## 3. Functions of Lysyl Oxidases in Tumour Progression and Metastasis

**Figure 4.**
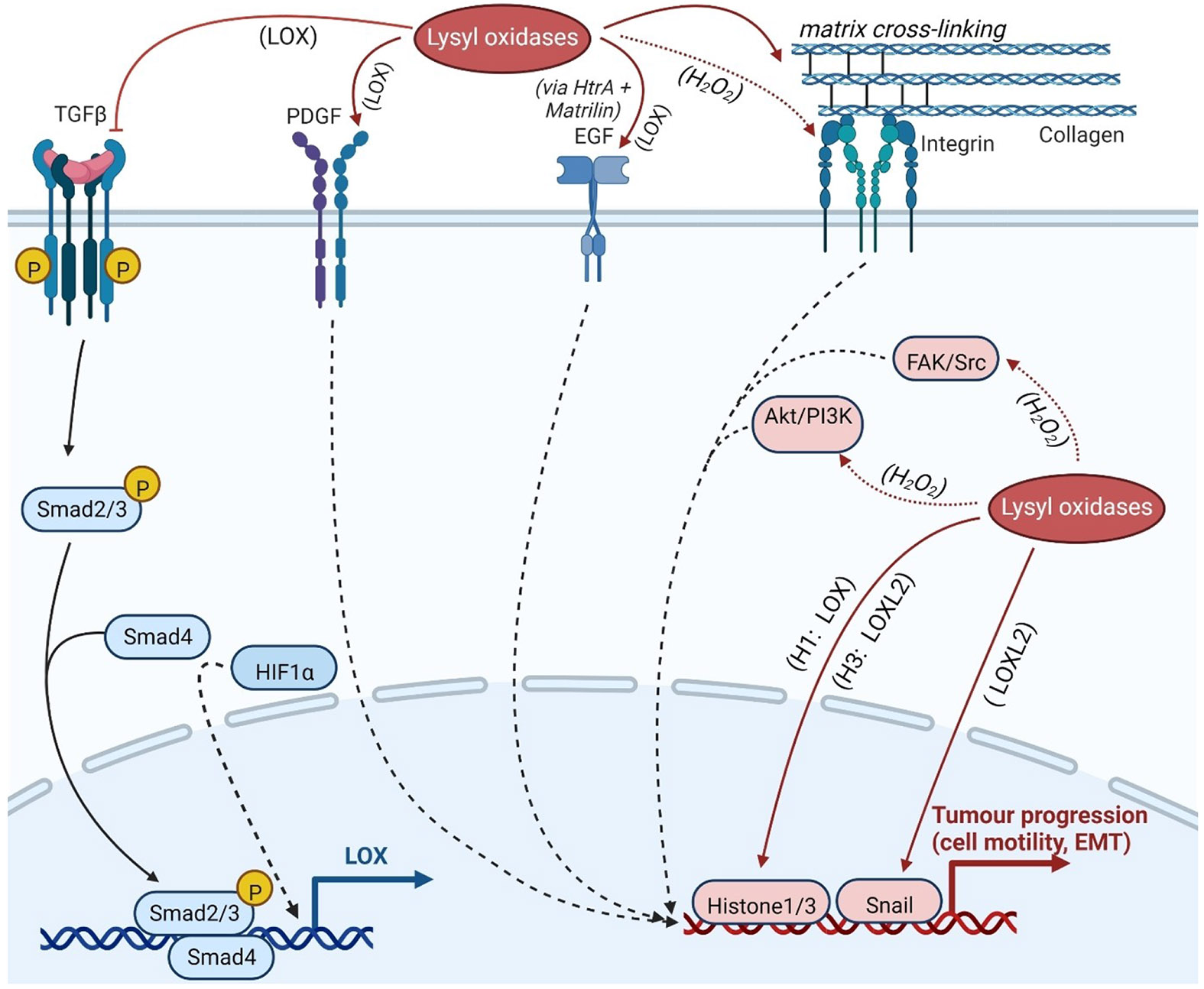
Intra- and extracellular functions of the lysyl oxidases LOX and LOXL2 in the context of tumour progression. Further explanation is included in the text. In the case of dashed arrows, the interaction partners of the signal transduction cascades have been omitted in favour of clarity. Created with BioRender.com.

Initial findings linking lysyl oxidases to neoplasia suggested that LOX was a tumour suppressor.^[48]^ However, the anti-oncogenic effects could be clearly attributed to interactions that are mediated by the propeptide domain of LOX independently of the oxidase activity.^[38b,49]^ In particular, the interactions of the LOX proenzyme with c-Raf and the c-Raf chaperone Hsp70 suppress the oncogenic RAS signalling pathway.^[50]^ In contrast, increased expression of lysyl oxidases has been associated with increased migration and invasive potential as well as increased capacity for epithelial-to-mesenchymal transition (EMT) of tumour cells.^[50c,51]^ Furthermore, there are numerous reports of increased expression of lysyl oxidases in tumour tissues, particularly the LOX and LOXL2 isoforms (Table 2). The mechanisms by which increased expression of these isoforms support tumour progression are complex and are influenced by numerous feedbacks in the context of extracellular and intracellular interaction and signal transduction networks. A number of review articles addressing the functions of lysyl oxidases in tumours in detail have been published in recent years, and are referred to here.^[30,52]^ The essential molecular mechanisms and major interaction partners through which LOX and LOXL2 contribute to the growth and spread of tumours will be summarised briefly in excerpts below and are illustrated schematically in Figure 4.

### 3.1. Action on extracellular substrates

A key regulator of cell-matrix interactions is the cytokine transforming growth factor (TGF)-β, which controls both the extracellular deposition of collagens and other ECM components and enzymes that catalyse their modification and degradation.^[71]^ The intracellular signal transduction cascade triggered by the TGF- receptor complex ultimately leads to the activation of Smad-type transcription factors, initiating the expression of LOX. Following secretion and activation of LOX in the extracellular milieu, it mediates oxidative cross-linking of collagen strands. Most likely, TGF-β itself is a substrate of LOX (pI > 8), which is deactivated by oxidative deamination of lysine residues at its C-terminus.^[72]^ This creates a mechanism of negative feedback on LOX expression.

This feedback appears to be largely overridden in the context of tumour progression.^[52h]^ Among other reasons, this is due to the fact that cells in solid tumours develop hypoxia once growth of the neoplastic tissue outstrips oxygen supply,^[73]^ which leads to the activation of the hypoxia inducible factor (HIF)-1. Since the LOX gene has a hypoxia-responsive element in its promoter, this also results in the upregulation of LOX expression.^[74]^ Although the increased activity of LOX under oxygen deprivation has been known for a long time,^[75]^ it was only pointed out in connection with cancer development in 2006 in a highly regarded paper,^[76]^ which, however, recently had to be withdrawn due to improper processing of Western blot images.^[77]^ Nevertheless, the conclusions drawn from this work stand, as they have been confirmed by subsequent work, including that of other groups.^[74,78]^ Similarly, expression of the lysyl oxidase isozymes LOXL2^[79]^ and LOXL4^[80]^ is under transcriptional control of HIF-1. Increased LOX activity under hypoxic conditions leads to increased mechanical strength of the ECM through increased oxidative cross-linking of collagen, resulting in increased mechanically stimulated mobilisation of TGF-β. In addition to TGF-β, another non-matrix protein, the platelet-derived growth factor (PDGF) receptor, was identified as a LOX substrate, whereby its residue Lys 162 is presumably oxidised to allysine. This post-translational modification of the receptor protein significantly increases its affinity for its ligand PDGF,^[72]^ which is an important mitogen in the context of tumour progression.^[81]^ A corresponding effect on the PDGF receptor of cancer-associated fibroblasts (CAFs) was also identified for LOXL2 secreted by tumour cells.^[82]^

Furthermore, the extracellular hydrogen peroxide released in the course of lysyl oxidase activity leads to activation of the phospahtidylinositol-3-kinase-Akt/protein kinase B (PI3 K-Akt/PKB) pathway, which in turn increases the expression of HIF-1 at the transcriptional level.^[83]^ Thus, LOX activity in turn amplifies the signalling pathways triggered by hypoxia. Another oncologically relevant kinase activated by hydrogen peroxide is the tyrosine kinase Src, which in turn activates the focal adhesion kinase (FAK).^[53b,61]^ Furthermore, integrins have been found to be activated by the formation of an intrachain disulfide bridge within the α-subunit, which in turn is induced by hydrogen peroxide.^[84]^ In this way, both LOX and LOXL2 support tumour progression.^[85]^

In addition, cellular signal transduction processes are influenced by the cross-linking of collagen strands triggered in the course of lysyl oxidase activity, which is essentially mediated by integrins of different subtypes.^[30]^ These cell adhesion proteins are significantly responsible for the interaction between cells and the ECM.^[86]^ Oxidative cross-linking of matrix proteins leads to increased mechanical strength, which stabilises the integrin complexes, while these are subject to increased internalisation on soft substrates.^[87]^ In turn, the strength of the ECM influences the expression and activity of lysyl oxidases. Thus, the stiffness of the adhesive substrate correlates with the expression of lysyl oxidases, especially LOXL2 in hepatocarcinoma cells, which could be attributed to the activation of the integrin β1/α5/JNK/c-JUN signalling pathway in these cells.^[88]^ Increased ECM strength has a similar effect on the expression of LOX in tumour-associated fibroblasts.^[30]^ Accordingly, lysyl oxidase activity and integrin signalling pathways mutually reinforce each other in the sense of a positive-feedback loop, and mechanical stiffening of the ECM contributes significantly to tumour malignancy.^[30,89]^

**Figure 5.**
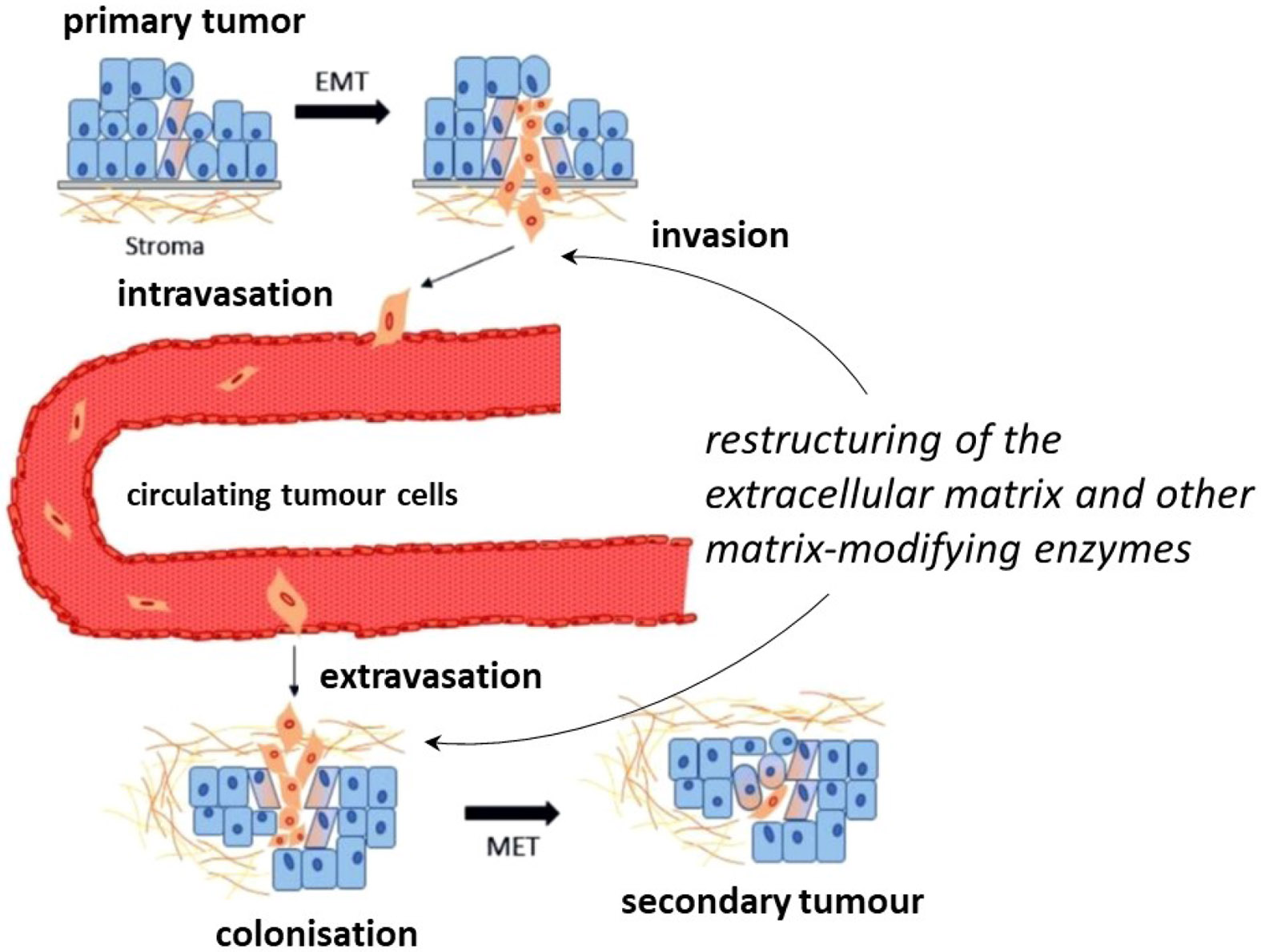
Functions of lysyl oxidases in the schematised process of tumour metastasis (EMT: epithelial-mesenchymal transition; MET: mesenchymal-epithelial transition). Adapted from Jones *et al.*^[52g]^ with permission, Copyright © 2020, Wiley-VCH Verlag GmbH & Co. KGaA, Weinheim (Germany).

This is impressively expressed in the context of tumour metastasis on the basis of the formation of the pre-metastatic niche. As early as 1889, the English surgeon Stephen Paget postulated in the context of his ground-breaking seed-and-soil hypothesis that conditions in the target tissue favouring tumour growth lead to organ-selective metastasis.^[90]^ In this sense, the colonisation of organs remote from the site of the primary tumour with cancer cells is preceded by local changes in the microenvironment of the target tissue, which are referred to as (pre)metastatic niches.^[91]^ At these sites, a massive remodelling of the tissue structure takes place, which is essentially induced by secretory factors of the cells of the primary tumour tissue. The latter enter the target tissue by extravasation, overcoming the endothelium from the blood (Figure 5). However, extracellular proteins secreted into the blood by the primary solid tumour as a result of pro-inflammatory stimuli and hypoxic conditions are equally important contributors to the formation of the pre-metastatic niche. In addition to growth factors and pro-oncogenic chemo- and cytokines, the secretory factors mainly include matrix-modifying enzymes, with lysyl oxidases appearing to play a key role.^[92]^ The interaction of all these factors modifies and restructures the ECM of the target tissue to create a growth-promoting metastatic niche in which an inflammatory and immunosuppressive microenvironment prevails.

Besides the isolated proteins mentioned above, exosomes play an important role in the formation of the pre-metastatic niche. These are extracellular vesicles that are detached from the cell membrane by budding and range in diameter from 30 to 150 nm. Exosomes are filled with a variety of molecules, including cytokines, growth factors, ECM components, nucleic acids, especially non-coding miRNAs, and lipids.^[93]^ Among others, hypoxic conditions influence the composition of exosomes, as demonstrated using glioma cells, whose exosomes exhibited increased lysyl oxidases and matrix metalloproteases under hypoxia.^[94]^ Furthermore, TGase 2, another matrix-modifying enzyme, was also identified as a component of exosomes,^[95]^ suggesting that these subcellular particles have an essential function in ECM remodelling. Lysyl oxidases secreted by solid primary tumours and transported to the target tissue with the blood have been shown to provide a tumourpromoting microenvironment in the affected organs. For example, in breast cancer mouse models (syngeneic 4T1 model^[96]^ and transgenic model derived from the cell lines MDA-MB-231/435^[80]^), it was shown that the isoforms LOX, LOXL2 and LOXL4 secreted by breast carcinoma cells enhance the cross-linking of collagen in the target tissue of the lung, which in turn facilitates the settlement of CD11b-positive bone marrow cells.^[97]^ These cells release the protease MMP-2 and other proangiogenic factors, which promote tumour cell invasion and growth.^[92]^ A similar function has been identified for LOX in the formation of bone metastases by breast cancer cells.^[98]^ Here, the enzyme secreted by the primary tumour causes increased osteolysis by activating or inhibiting osteoclasts and osteoblasts, promoting metastatic colonisation of bone tissue. The prometastatic effect of LOX was thereby suppressed by the inhibitor β-aminopropionitrile, highlighting the importance of its catalytic function in this process.^[92]^ This compound was also used in another study on the function of lysyl oxidases in metastasis in the breast cancer mouse model (MDA-MB-231), from the results of which it could be concluded that lysyl oxidase activity is also important in the process of extravasation of tumour cells from the bloodstream.^[99]^ Thus, the restructuring of the ECM triggered by the activity of lysyl oxidases is essential both for the invasion of primary tumours as well as for the extravasation and colonisation of distant organs during the process of metastasis (Figure 5).

In addition to the activity of lysyl oxidases in the target tissue, metastasis is also supported by their activity in the solid primary tumour. In this process, lysyl oxidase-mediated cross-linking of the tumour-associated ECM leads to the enhancement of focal contacts involving integrins and, as a consequence, activation of the PI3 K-Akt pathway, which considerably increases the invasive potential of cancer cells, as has been shown for various tumour entities.^[30,92]^ In this context, lysyl oxidases are apparently not expressed by the neoplastic cells alone, but also by the benign cells of the associated stroma. Likewise, in the sense of such cooperation between malignant and benign cells, the collagen cross-linking triggered by tumour lysyl oxidases can lead to integrin-mediated activation of CAFs. A further pro-tumorigenic and pro-metastatic function arises through their involvement in angiogenesis. Secretion of LOXL2 by tumour-associated endothelial cells has been shown to support neovascularisation of the neoplastic tissue in murine models of SKOV-3 ovarian cancer and Lewis lung carcinoma.^[100]^ These findings highlight that lysyl oxidases are key enzymes in hypoxia-induced tumour metastasis.^[101]^ It should be also mentioned, that lysyl oxidase-mediated stiffening of the ECM, as in occurs in the fibrotic response of tissue to pathogenic stimuli, in concert with chronic inflammation and other oncogenic processes is considered a driver of carcinogenesis, as reflected in the term “precancerous niche”.^[102]^ Distinct functions of lysyl oxidase isozymes in both the primary tumour and secondary metastatic sites were recently reviewed.^[103]^

### 3.2. Action on intracellular substrates

Besides the effect on extracellular proteins, the oxidative modification of soluble, intracellularly localised proteins catalysed by lysyl oxidases is also associated with tumour progression. However, much less is known in this regard compared to the functions of lysyl oxidases outside the cell. One soluble intracellular protein whose activity has been shown to be controlled by LOXL2 is the transcription factor Snail. This is a key protein of EMT. Unlike other EMT-inducing transcription factors, Snail is constitutively expressed at a high rate of synthesis, but is subject to rapid degradation. This is induced by glycogen synthase kinase (GSK)-3β-catalysed phosphorylation, resulting in ubiquitinylation and proteasomal degradation of Snail. One mechanism for stabilising the Snail protein is its interaction with LOXL2, which probably leads to oxidative deamination of lysine residues 98 and 137, presumably resulting in a conformational change such that phosphorylation can no longer proceed.^[104]^ In this study, the overexpression of LOXL3 was also shown to interact with Snail, although the pathobiological significance remains to be further elaborated. The effect of Snail as a transcription factor leads, among other processes, to the repression of the adhesion protein E-cadherin, which mediates the adoption of a mesenchymal phenotype and thus the invasive capacity of the tumour cell.^[105]^

The suppression of E-cadherin is also caused by LOXL2-mediated modification of another intracellular protein. It has been shown that the activity of LOXL2 oxidatively removes the trimethylammonio group of the lysine residue triple methylated at the ε-amino group in position 4 of histone H3 (H3 K4me3) to form an allysine residue. Oxidation of the *N*-mono- and *N*,*N*-dimethylated proteins (H3 K4me and H3 K4me2, respectively) does not take place, as demonstrated by mass spectrometry.^[106]^ Due to the lack of nucleophilicity of the *N*,*N*,*N*-trimethylated lysine residue, this modification is not compatible with the catalytic mechanism of the lysyl oxidases described in the previous section. However, the formation of the allysine-containing histone protein was reliably detected by IR spectroscopy^[106-107]^ and by detection with biotinyl hydrazide and streptavidin.^[108]^ Therefore, it can apparently be assumed that first a hydrolytic release of trimethylamine occurs with the formation of ε-hydroxynorleucine, which is subsequently dehydrogenated to allysine. Since water occurs as a reactant in lysyl oxidase-catalysed reactions in any case, it is conceivable that the hydrolysis of the C─N single bond to the corresponding ε-hydroxynorleucine could be eventually catalysed by LOXL2. The subsequent conversion of the primary alcohol to the aldehyde would be possible in principle, since reactivity of the electrophilic carbonyl group of the LTQ cofactor is also conceivable towards hydroxy groups as nucleophiles and this could proceed mechanistically in analogy to the oxidation reactions catalysed by pyrroloquinoline quinone (PQQ)-dependent alcohol dehydrogenases.^[109]^ However, further enzymological studies are needed to confirm these conjectures. Oxidative modification of H3 K4me3 results in the repression of the CDH1 promoter, which represses transcription of the E-cadherin-encoding CDH1 gene.^[110]^ Therefore, LOXL2-mediated modifications of the histone H3 protein and those of Snail act synergistically with respect to tumour progression. In terms of removing the methyl groups from H3 K4me3, LOXL2 thus functions as an epigenetic eraser. Furthermore, it was demonstrated that the concentration of the oxidatively modified H3 protein is higher in triple-negative breast cancer cell lines than in other breast cancer types and that the siRNA-mediated knockdown of LOXL2 leads to the activation of the DNA damage response and enhanced sensitivity towards cytostatic agents in these cell lines.^[108]^

LOX has also been associated with the modification of histone proteins, particularly histone H1, which interestingly shares the AAKAAA sequence with the confirmed LOX substrate tropoelastin.^[37b,111]^ LOX-catalysed turnover of histone H1 was demonstrated in vitro.^[37b]^ In MCF-7 cells, desmosine and isodesmosine cross-links for this protein could be detected in dependence on LOX expression, although not directly by structural analysis but by immunocytochemical detection.^[112]^ Furthermore, evidence for the oxidative dimerisation of H1 could be derived within the same study. Oxidative deamination and subsequent oligomerisations of histone H1 lead to increased promoter activity in the genomically integrated DNA of mouse mammary tumour virus (MMTV) in MMTV-transfected MCF-7 cells^[112]^ and to chromatin decondensation in COS-7 and NRK-49F cells.^[113]^ LOX-mediated modification of nuclear proteins is consistent with results on translocation of extracellular LOX into the nucleus^[47]^ as mentioned above.

Other lysyl oxidase isozymes also appear to play a role in the modification of intracellular proteins. In particular, evidence exists that LOXL3 is capable of removing acetyl groups from Lys-acetylated STAT3, which can take place both hydrolytically and oxidatively in the sense of deacetamidation. Interestingly, the centre of the hydrolytic deacetylase activity could be located in the N-terminal SRCR domain of LOXL3. With respect to oxidative deacetamidation, the acetylated compared to the non-modified STAT3 protein is preferentially converted to the allysine-containing product, which is an extremely interesting enzyme-chemical finding which again raises questions regarding the catalytic mechanism.^[114]^ The transcription factor STAT3 can be activated by both phosphorylation and acetylation, which triggers processes of cell proliferation and differentiation, the latter especially in the case of immune cells. Thus, intracellular LOXL3 possibly exerts a tumour suppressive effect, while the activity of this enzyme in the context of tumour-associated inflammatory processes could possibly promote tumour progression.^[114-115]^ These findings show that lysyl oxidases appear to have an important function in the post-translational modification of intracellular proteins, also with regard to epigenetic regulation processes.

Even though the isozymes LOXL1 and LOXL4 have not been in the focus in connection with cancer for a long time, recent results also suggest an involvement of these lysyl oxidases in tumour progression, which was summarised in a recent review.^[52k]^ Interestingly, a strong overexpression of LOXL4 at the mRNA level has been observed in triple-negative breast cancer.^[116]^ Recently, it was shown that both mutation of LOXL4 to the catalytically inactive protein and genetic ablation of the entire protein in MDA-MB-231 breast cancer cells lead to significantly reduced tumour growth. The enhancing effect of LOXL4 on tumour growth and invasion could be attributed to the oxidative cross-linking of annexin A2, which in turn prevents the internalisation of integrin-β1.^[117]^

### 3.3. Brief overview on the functions of lysyl oxidases in other disease processes

As mentioned above, besides cancer, lysyl oxidases have emerged as important targets in cardiovascular homeostasis and disease. The central importance of LOX and LOXL2 in vascular development is clearly illustrated in the failure of the proper development of the cardiovascular system in mice with germline knockouts of these enzyme, and high perinatal lethality due to rupture of the diaphragm or aortic aneurysms.^[67,118]^ In response to hypertension, the blood vessels undergo remodelling in order to be able to withstand the elevated mechanical load imposed upon them, failing which, aortic aneurysms arise. In mice with experimental hypertension induced by angiotensin II infusion, lysyl oxidases stabilise aneurysms,^[119]^ and inhibition of lysyl oxidase using β-amino-propionitrile (βAPN) leads to a marked increase in the formation of aneurysms that are more prone to rupture, such that βAPN is used to induced experimental aortic aneurysms in mice.^[120]^ Thus, in the context of development and hypertension induced aneurysms, lysyl oxidases are necessary for cardiovascular homeostasis. The detrimental effects of lysyl oxidase activation are illustrated in aging, where loss of nitric oxide bioavailability results in higher LOXL2 levels in the ECM of the aorta. The specific role of LOXL2 in promoting the mechanical decline of the vasculature during aging was identified using mice in which LOXL2 expression was depleted.^[118a]^ In addition, LOX and LOXL2 are both involved in fibrosis in the heart and lung, as reviewed elsewhere.^[52d,121]^

The functions of lysyl oxidases in tumours as outlined above show that they appear to be key enzymes for tumour progression and metastasis, which makes the development of inhibitors and imaging probes for tumour therapy and their non-invasive detection in vivo, respectively, highly attractive. Therefore, recent inhibitor development will be highlighted in the next section followed by an overview on lysyl oxidase-directed imaging probes.

## 4. Development of Lysyl Oxidase Inhibitors

The above-mentioned βAPN, by far the most commonly used inhibitor, is already known from the early years of lysyl oxidase research, as its effect on the structure of connective tissue led to the discovery of these enzymes.^[122]^ For a long time, the development of lysyl oxidase inhibitors was not the focus of the work of medicinal chemistry research groups, both in academia and industry, which was probably due in part to the fact that the pathophysiological significance of these enzymes was initially largely unknown and structure-based drug development was hampered by the lack of knowledge of the enzyme structures. The investigation of inhibitor compounds on LOX was primarily carried out by the working group around Kagan, whereby this work seemed to be more motivated by the elucidation of the catalytic mechanism than by pharmacological applications.^[123]^ Primary amines were identified as substrateanalogue inhibitors that form “dead end” complexes with the LTQ cofactor, i. e. the amino group condenses with the cofactor to form the quinone semi-imine, but the further steps leading to the releases of the aldehyde product do not take place. In addition to β-aminopropionitrile as the prototypical lysyl oxidase inhibitor, 2-haloethylamines and 2-nitroethylamines, homoserine lactone and its sulfur and selenium analogues as well as benzylamine derivatives belong to this category. The latter compounds seem to act as alternate substrate-based inhibitors, since the *k*_cat_-values for *para*-substituted benzylamines decreased with increasing Hammett σ_p_-substituent constants, which indicates stabilisation of the aminophenolate-aldimine intermediate originating from deprotonation of the initially formed quinone semi-imine.^[13b]^ Vicinal diamines appear to be particularly interesting as inhibitor compounds, since they undergo cyclocondensation with both carbonyl groups of the quinone cofactor with irreversible formation of the corresponding enzyme-bound quinoxaline.^[124]^ Furthermore, hydrazine derivatives such as semicarbazide base their inhibitory effect on the carbonyl reactivity towards the cofactor of the lysyl oxidases, whereby representatives of this class of compounds often also inhibit TPQ-dependent amine oxidases with sometimes higher potency.^[125]^

### 4.1. Bioreductively activatable βAPN prodrugs

**Figure 6.**
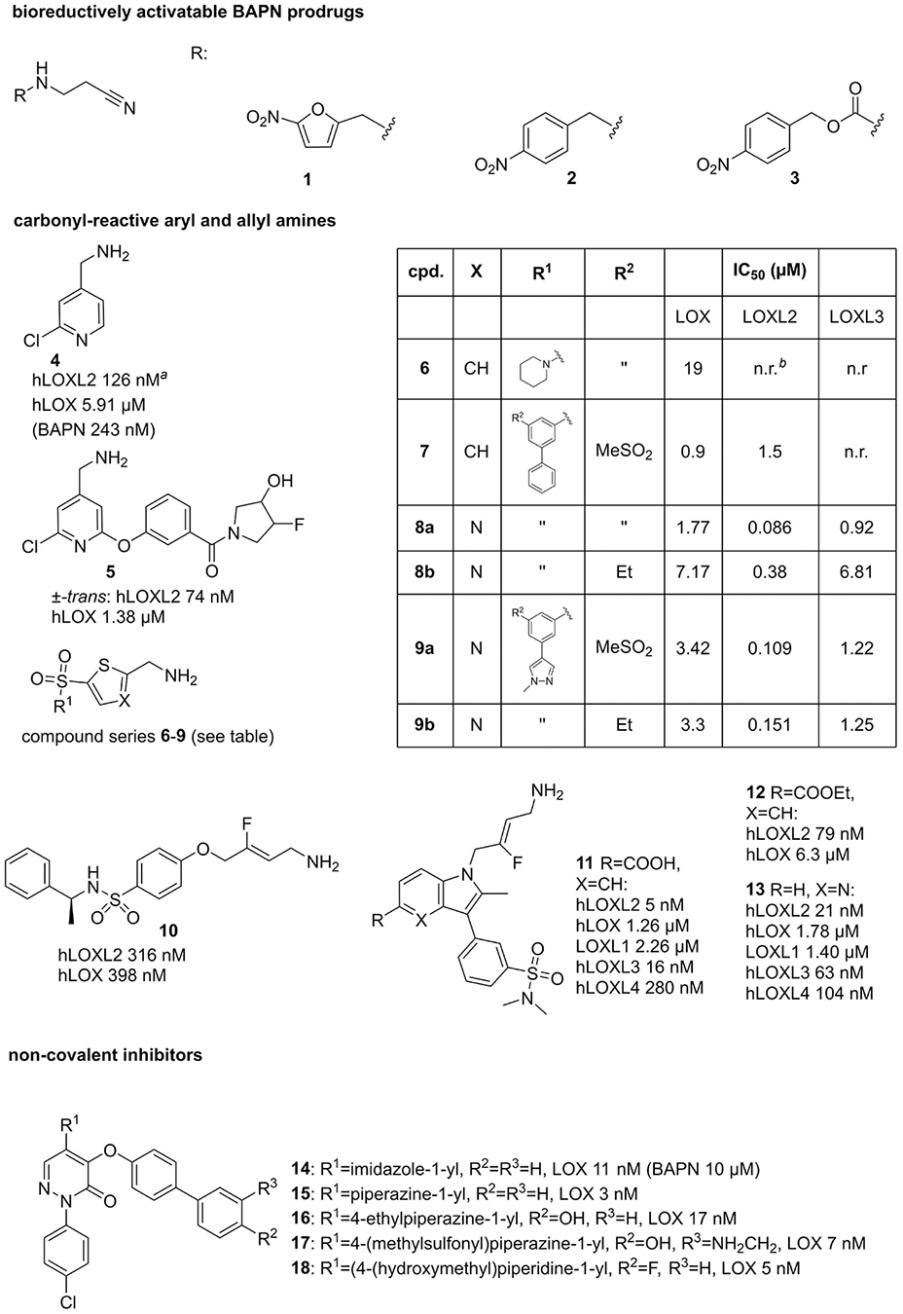
Newer inhibitor compounds (as of 2003) of lysyl oxidases. ^*a*^ All reported inhibitory activities represent IC50 values; ^*b*^ not reported.

With regard to potential applications of first-generation lysyl oxidase inhibitors in the therapy of tumour diseases, reductively activatable βAPN prodrugs deserve special mention. Considering the relationship between hypoxia and LOX expression, *N*-nitroaryl derivatives of βAPN were synthesised by Granchi *et al.* and their antineoplastic activity was investigated in MDA-MB-231 breast cancer cells.^[126]^ They exploited the bioreduction of nitro to hydroxylamino groups known for nitroarenes under reduced oxygen partial pressure (< 5-15 mbar),^[73]^ whereby the βAPN derivatives undergo spontaneous 1,6-elimination with release of the inhibitor. For compounds **1** and **3** (Figure 6), the difference between inhibitory activity under normoxic and hypoxic conditions was particularly striking for both the determination of lysyl oxidase activity in conditioned medium and in the cell invasion assay. In addition, a correlation between inhibitory activity under hypoxia with standard potentials determined by cyclovoltammetry was indicated, as compound **1** with the least negative redox potential of −610 mV exhibited the greatest hypoxia selectivity.^[126]^

### 4.2. Hetarylmethylamine-based inhibitors as time-dependent reversible inhibitors

#### 4.2.1. Pyridylmethylamines

Kagan’s pioneering work on amine-based substrate analogue inhibitors formed the basis for more recent developments of lysyl oxidase inhibitors (Figure 6). Taking up the study of Williamson and Kagan on substituted benzylamines mentioned above, a paper published in 2017 reports on investigating the inhibitory activity of such compounds towards LOX and LOXL2, with those compounds with electron-withdrawing substituents in the 4-position proving particularly potent. The isoelectronic replacement of the phenyl residue with 4-pyridyl led to a further increase in activity. Compound **4** was identified as the most potent derivative towards LOXL2, inhibiting LOXL2 by a factor of 31 more strongly than LOX. Apparently, the corresponding LTQ-aldimine adduct is particularly stabilised by conjugation with the electron-deficient pyridine ring, resulting in a quasi-irreversible inhibition.^[127]^

Further exploration of structure-activity relationships for picolylamine-based inhibitors resulted in compounds exhibiting inhibitory activities (IC_50_ values) towards LOXL2 in the doubledigit nanomolar range with even greater selectivity for LOXL2 over LOX and no detectable inhibitory activity for other amine oxidases, such as compound **5** (Figure 6). This inhibitor showed furthermore favourable pharmacokinetic properties in rats and mice, which allowed for investigating its activity in the mouse model of bleomycin-induced pulmonary fibrosis. Daily oral administration of **5** at a dose of 30 mg/kg for 15 days resulted in a significant reduction in fibrotic tissue.^[128]^

#### 4.2.2. Thenylamines

LOX inhibitors with similar inhibitory mechanism were found in 5-sulfonyl substituted 2-thienylmethylamines (also referred to as thenylamines) by Leung et al.^[129]^ Here, the lead compound **6** (Figure 6) was identified by high-throughput screening. Targeted structural variations led to compound **7**, which inhibits LOX with an IC_50_ value of 900 nM in the absence of selectivity with respect to inhibition of LOXL2. However, the inhibitory effect towards lysyl oxidases is selective compared to other amine oxidases. In analogy to the 4-picolyamines, the sulfonyl residue in position 5 of the thiophene ring leads to electronic stabilisation of the LTQ-aldimine adduct formed after tautomerisation of the quinone semi-imine. The favourable pharmacokinetic properties (stability, bioavailability) of inhibitor **7** allowed its in vivo evaluation in a mouse model of metastatic breast carcinoma. Daily oral administration at a dose of 70 mg/kg for 20 days^[130]^ resulted in a significant reduction in both the number and area of lung metastases compared to control treatment.^[129]^

#### 4.2.3. Thiazolylmethylamines

**Figure 7.**
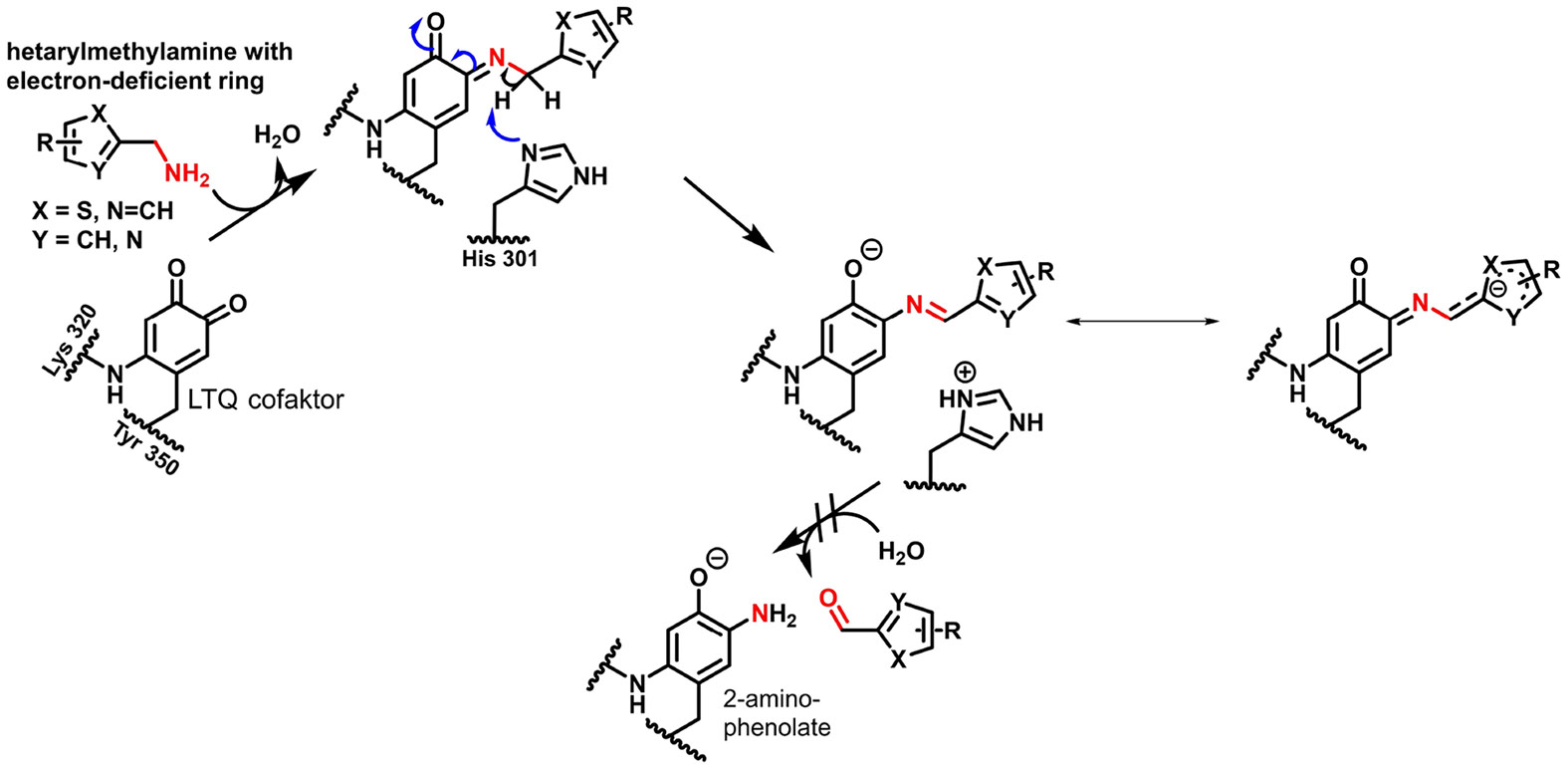
Postulated mechanism of lysyl oxidase inhibition by hetarylamethylamine-based inhibitors.

In the course of the study by Leung et al., analogues containing five-membered hetarenes other than thiophene were synthesised, among which a 2-aminomethyl-5-sulfonylthiazole derivative largely retains activity of the thiophene-containing parent compound. In contrast, the analogue with the regioisomeric 5-aminomethylene-2-sulfonylthiazole substitution pattern but otherwise identic constitution exhibited strongly diminished inhibitory potency with IC_50_ values in the two-digit micromolar range towards LOX and LOXL2.^[129]^ Further structure-activity exploration of the 2-aminomethylthiazole core regarding inhibitory activity towards LOXL2 has revealed that compound **8a** (Figure 6), the 3-aza analogue of thiophene **7**, showed slightly increased potency, which was attributed to resonance effects due to the decreased electron density of the thiazole ring and/or additional non-covalent interactions conferred by the ring nitrogen atom. Notably, attaching electron-withdrawing substituents such as fluorine and chlorine to the position identical to that of the thiophene ring resulted in decreased potency. The importance of the arylsulfonyl residue in 5-position of the thiazole ring was confirmed, however, a decreased oxidation state of the bridging sulfur atom is less detrimental than in the 2-aminomethylthiophene series. Similarly, influence of the substitution site for the arylsulfonyl moiety was less pronounced in the thiazole series, as 2,4- and 2,5-substituted thiazoles showed less different inhibitory activities compared to their thiophene-containing counterparts. Exploration of structure-activity relationships for the terminal phenyl ring suggested that the influence of the electron-withdrawing effect of the arylsulfonyl on the formation and stability of the enzyme inhibitor complex is somewhat blunted in the context of the electron-deficient thiazole ring. On the basis of the SAR obtained for the 2-aminomethylthiazoles and 2-aminomethylthiophenes for inhibition of LOXL2, predictive CoMFA-related 3D-QSAR and visual qualitative models were developed, which – among other pharmacophoric traits – predicted a sterically restricted 3-position of the aminomethyl-substituted heterocycle requiring negative partial charge. Investigations of the mode of inhibition proved that the enzyme-inhibitor interaction is time-dependent since the IC_50_ values decreased with increasing preincubation times. Furthermore, monitoring of the inhibitory activity in jump-dilution assays suggest that thiazole **9a** acts as a slowly reversible inhibitor since the recovery of activity following dilution was 30% compared the DMSO control, while 80% of activity were regained in the case of compound **7**. Therefore, on the basis of these results it can be hypothesised that those hetarylaminomethylamines act by forming stabilised aminophenol aldimines, for which hydrolytic release of the aldehyde products is blocked because nucleophilic attack of the C─N double bond is hindered for electronic and/or steric reasons (Figure 7). Release of the free enzyme from the enzyme-inhibitor complex might occur by reprotonation leading back to the quinone semi-imine, from which the LTQ cofactor is restored by hydrolysis under the release of the inhibitor molecule. However, it should be clarified whether the original inhibitor is still unmodified after dilution or partial modification to the corresponding aromatic aldehyde occurred to support this assumption. Regarding inhibition of different LOX isoforms, compounds **8a**, **8b**, **9a**, and **9b** showed ≥ 10-fold selectivity toward LOXL2 over LOX and LOXL3. Even higher LOXL2 selectivity is achieved within the 2,4-disubstituted thiazole series (compounds with 2,4-susbtituted thiazole ring and otherwise identical substituents to compounds **8a**, **9a** and **9b**). Highest inhibitory activity towards LOXL3 was exhibited by compound **8a**, which still is about 10-fold more potent toward LOXL2 and only slightly less active towards LOX. Furthermore, good selectivities were observed with regards to inhibition of monoamine oxidases MAO─A and MAO─B and diamine oxidase. Notably, the selectivity over plasma amine oxidase is greater for the pyrazole-containing aminomethylthiazoles **9a** and **9b** than for the thiophene-based inhibitor **7**. The pharmacokinetic properties of selected thiazoles were also more favourable in terms of plasma exposure upon oral administration and metabolic stability against rat liver microsomes compared to compound **7**. In particular, compound **9b** was found to exhibit an oral bioavailability of 68% and a maximum plasma concentration (C_max_) of 26.7 μM upon administration of a single dose of 50 mg/kg into mice, which was therefore selected for assessing the antitumour efficacy in vivo. For that purpose, a LOX-driven spontaneous murine breast cancer model was established.^[130]^ The tumour-bearing mice were daily dosed with inhibitor **9b** at 70 mg/kg over 34 days, starting at the time when tumours became palpable, which was approximately 60 days after birth. Tumour growth in the treatment group (*n*=3) was significantly delayed so that ethically limited maximum tumour volumes were not reached, whereas in the control group (*n*=5) all animals had to be euthanised because the limiting tumour volumes were reached.^[131]^

### 4.3. 3-Fluoroallylamines as irreversible inhibitors

Another recently reported class of lysyl oxidase inhibitors are the 3-fluoroallylamines, which are in a sense related to β-aminopropionitrile, insofar as its ketene imine tautomer is considered. Fluoroallylamines are known as inhibitors of both TPQ- and flavin-dependent amine oxidases, such as mofegiline as an inhibitor of monoamine oxidase B.^[132]^ Like the presented hetaryl methylamines, fluoroallylamines bind in a substrate-analogous manner. In the case of TPQ-dependent enzymes, after formation of the aldimine adduct, attack on the C-3 of the fluoroallylamine by a nucleophilic residue in the vicinity occurs, which results in substitution of the fluorine atom due to an addition-elimination mechanism.^[133]^ Based on known compounds with 3-fluoroallylamine partial structure, compound **10**^[134]^ (Figure 6) was first identified as a LOX inhibitor without selectivity over LOXL2, with the *Z* isomer shown to be about an order of magnitude more active than the *E* isomer. The attachment of aromatic and heteroaromatic residues and focussed structural variation at the 4-position led to compound **11**,^[135]^ which exhibited inhibitory activity towards LOXL2 in the single-digit nanomolar range (IC_50_ = 5 nM), whereas among the other four lysyl oxidase isozymes, the inhibitory activity towards LOXL3 was strongest with 16 nM (IC_50_). Compound **11** proved to be an irreversible inhibitor, with a second-order inactivation constant (*k*_inact_/*K*_I_) of 178,715 M ^−1^s^−1^, as calculated from the reported parameters *k*_inact_ and *K*_I_. Removal of the fluorine atom leads to reversible inhibition. The compound was further shown to be sufficiently metabolically stable *in vivo.* However, it is poorly bioavailable after oral administration due to its zwitterionic structure. Therefore, ethyl ester **12** was prepared, which is able to significantly reduce the extent of fibrotic tissue after oral administration at a dose of 5 mg/kg in both the case of CCl_4_-induced liver fibrosis and the model of bleomycin-induced lung fibrosis (each murine models).^[135]^

The inhibitory activity of 5-azaindole analogue **13** (Figure 6), which was disclosed previously in a patent,^[136]^ was reported separately.^[137]^ The compound was investigated in the CCl_4_-induced liver fibrosis, which has revealed comparable results to compound **12**. Furthermore, compound **13** was evaluated in a physiologically more relevant model of streptotocin-induced non-alcoholic steatohepatosis. After oral administration of **13** at a daily dose of 10 mg/kg for a period of 6 weeks, histochemically assessed liver injury, including hepatocellular enlargement, was significantly reduced compared to the untreated control group. Compound **13** was also shown to be effective in reducing the fibrotic response in the context of myocardial infarction-induced pathological tissue remodelling.^[137]^

### 4.4. Pyridazin-3-ones as non-covalent inhibitors

Pyridazin-3-ones, which in contrast to the above-mentioned compounds do not have a primary amino group, were already disclosed as LOX inhibitors in a patent from Bayer in 2003 (compounds **14–18**, Figure 6).^[138]^ However, no information was provided on the inhibition mechanism and the developmental origin of this class of compounds. The pyridazinone structural element is also found in analgesic and anti-inflammatory agents such as the analgesic emorfazone.^[139]^ Derivative **14** inhibits bovine LOX with an IC_50_ value of 11 nM, while βAPN has a corresponding value of 10 μM in the identical assay. Furthermore, the antifibrotic effect of the compound was demonstrated in the bile duct ligation and CCl_4_-induced liver fibrosis models (rat as model organism in each case).^[138]^

The studies on the clinically motivated development of lysyl oxidase inhibitors reviewed above highlight the potential that inhibition of these enzymes offers with regard to the therapy of metastatic tumours and fibrotic diseases. Further studies on the clinical translation of these as well as the development of further inhibitor compounds are to be expected.

## 5. Development of Lysyl Oxidase-Targeted Imaging Probes

Considering the biomedical potential of the lysyl oxidases, molecular probes that enable the detection and imaging of these enzymes at the cellular or organismal level *in vivo* are desirable and several reports in this regard were published in the recent decade. Methods for activity determination of lysyl oxidases were reviewed recently.^[140]^ Emphasis in this section is placed on methods that allow for visualisation of lysyl oxidase activity at a microscopic and macroscopic scale.

### 5.1. Probes for fluorescence-, ELISA-, and western-blot-based detection of lysyl oxidases

**Figure 8.**
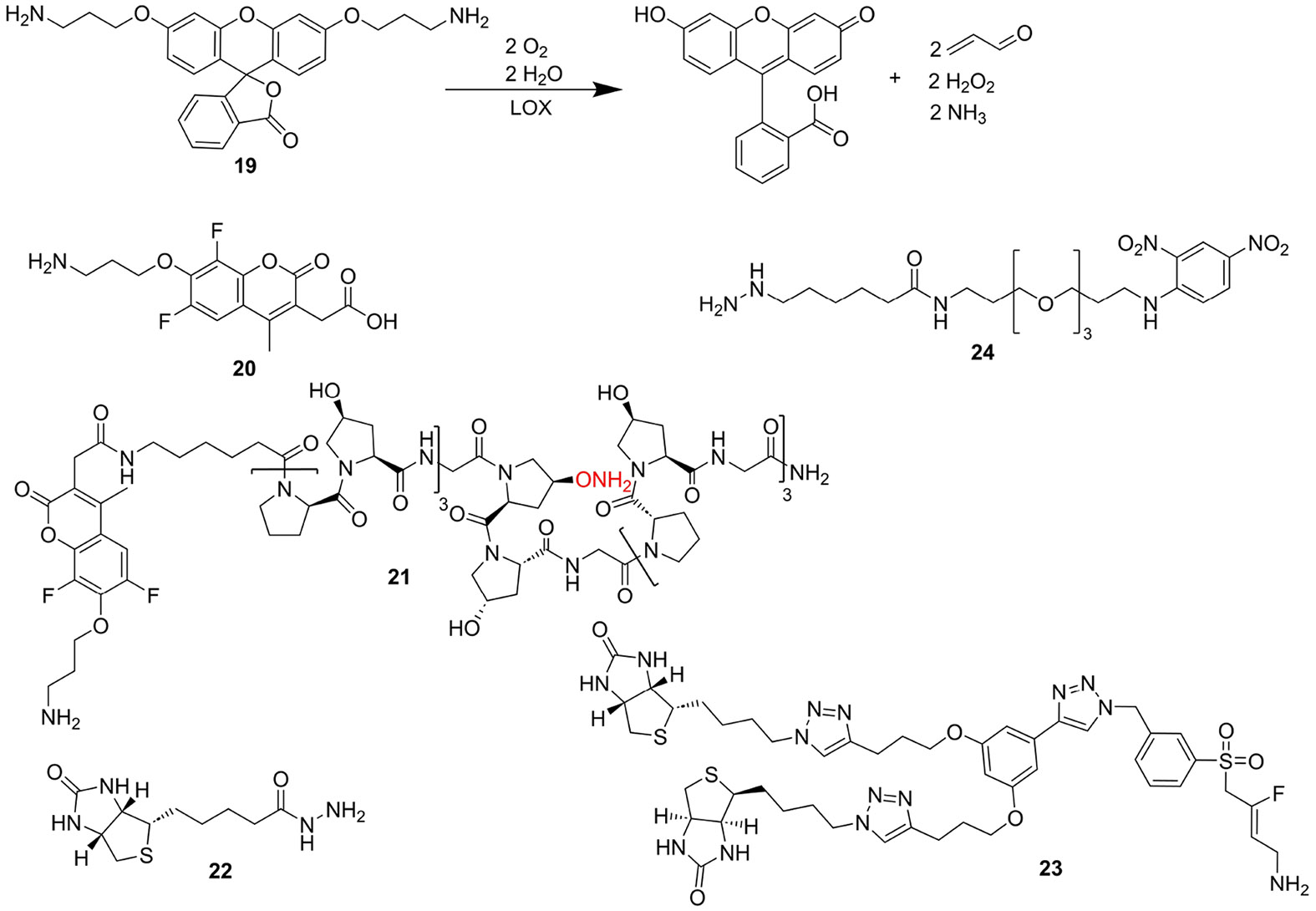
Probes for fluorescence-, ELISA- and Western blot-detection of lysyl oxidases.

A substrate-based probe that enables fluorescence-microscopic detection of catalytically active lysyl oxidases has been identified in 3,6-*O*,*O*’-bis-(2-aminopropyl)fluorescein (**19**).^[141]^ Compound **19** is converted as a “double” substrate to the corresponding dialdehyde, which in turn spontaneously undergoes double β-elimination to acrolein and free fluorescein (Figure 8). As a fluorogenic substrate, compound **19** is suitable for detecting lysyl oxidase activity (LOX and LOXL2) in lung tissue homogenate as well as in the tissue section of a fibrotic lung (donkey as model organism). Activation by monoamine oxidases, for which similar fluorogenic substrates have been described,^[142]^ does not occur. In the corresponding model, the compound could also be used for *in situ* visualisation of lysyl oxidase activity in lung tissue by fluorescence microscopy-coupled endoscopy.^[141]^

The principle of amine oxidase-mediated uncaging of 3-aminopropoxy-functionalised fluorophores has also been applied to less bulky systems. In particular, Aronoff *et al.* selected Pacific blue-derived 3-carboxymethyl-6,8-difluoro-7-hydroxy-4-methylcoumarin as fluorophore for designing a lysyl oxidaseresponsive turn-on fluorescent probe. Its 7-hydroxy group was etherified with the aminopropyl group (compound **20**, Figure 8) for caging its fluorescence.^[143]^ *O*-Alkylation resulted in a hypsochromic shift of the absorption maximum from 360 nm to 310 nm under concomitantly strongly diminished fluorescence quantum yield compared to the unfunctionalised dye, which allows for selective excitation of the unmasked dye. The capability of **20** for detecting lysyl oxidase activity in complex biological sample material was demonstrated for murine skin tissue homogenates. A total protein concentration as low as 0.1 mg/mL was sufficient to result in a 20-fold increase of fluorescence intensity relative to background. Pretreatment of the homogenate with βAPN at a concentration of 100 μM resulted in attenuation of the fluorescence signal down to a value that is similar as in the absence of sample material. Further correlation of probe activation to physiological variances of LOX activity with age and gender was established. Application of **20** for *ex vivo* imaging of lysyl oxidase activity by intradermal injection was hampered by diffusion and clearance of the probe and/or the uncaged fluorophore. To overcome this limitation, the probe was conjugated to a collagen-mimetic 21mer peptide containing (4*S*)-aminooxyproline, which allows for covalent trapping of the lysyl oxidase substrate by oxime formation with allysine sites in vivo. The peptidic moiety of the resulting compound **21** (Figure 8) is capable of self-assembly into triple helices, which facilitates incorporation into endogenous collagen strands. Hence, peptidic conjugate **21** can be considered as a probe with two heads for targeting lysyl oxidase, as both enrichment via oxime formation and triggering of fluorescence are mediated by lysyl oxidase, even though the former involves the enzyme indirectly through the prior formation of allysine. Microscopic analysis of tissue samples taken from mice 5 days after intradermal injection of **21** has revealed that the Pacific blue-borne fluorescence colocalised with fibrous extracellular structures aligned parallel to the dermal layer, which mainly consisted of type III collagen. Control probes with a peptidic moiety that lacked the aminooxyproline residue but otherwise were composed of the collagen-derived amino acid triplets (21mer peptide amides composed of (Pro-Hyp-Gly)_7_ and (Pro-Pro-Gly)_7_ N-terminally conjugated to **20**) behaved similar to the unconjugated caged fluorophore. On the other hand, if the peptidic part was reduced to isolated aminooxyproline amide, the blue fluorescence was unselectively distributed over the entire tissue section. The pivotal function of lysyl oxidases and collagen remodelling in tumour progression as outlined above motivated Aronoff *et al.* to investigate probe **21** by fluorescence microscopy in murine xenograft models derived from cell lines SCC13 and A431, which are originating from human squamous cell and epidermoid carcinoma, respectively. Remarkably, incubation of SCC13 tumour sections with **21** revealed that the probe accumulated at the edge of the tumour, while type I collagen was more or less uniformly distributed within the neoplastic tissue. Similar results were obtained for A431 tumour tissue.^[143]^

The aminooxyproline-containing fluorescent probe **21** partly exploits the unique enzymatic activity of lysyl oxidases generating protein-bound aldehyde groups for enrichment via oxime formation prior to lysyl oxidase-catalysed conversion to the fluorescent product. However, even probes which rely exclusively on carbonyl reactivity such as hydrazine and hydroxylamine derivatives are suitable for indirect imaging of lysyl oxidase activity. With respect to the former functional group, the utility of commercially available biotinylhydrazide for microscopic detection of allysine and derived cross-links was recently elaborated. For this purpose, A7r5 rat vascular smooth muscle cells, wild-type and virally transfected for expression of LOXL2 were incubated with 100 μM biotin-hydrazide (BHZ, compound **22** in Figure 8) for 24 h. After fixation, detection of the incorporated BHZ was done with fluorescein-conjugated streptavidin and cells were co-immunostained for LOXL2. Biotin- and LOXL2-detecting fluorescence signals colocalised well and intensity of the fluorescein-derived signal correlated with the LOXL2 levels of the LOXL2 and LOXL2-DM overexpressing cell lines, which served as control. The latter species represent LOXL2 double-mutant H626/628Q, whose copper-binding capability and therefore LTQ cofactor maturation is compromised. Treatment of the LOXL2-A7r5 cells with βAPN and LOXL2-selective inhibitor PAT-1251 (compound **5** in Figure 6) reduced the signal resulting from BHZ incorporation to approximately the levels of non-transfected and LOXL2-DM cells. Reaction of the BHZ with the carbonyl groups of the LTQ cofactor was ruled out by demonstrating that no enrichment of neither LOXL2 nor LOXL2-DM secreted into the culture supernatant of transfected cells occurred on streptavidin-conjugated agarose beads. Contribution of other aldehyde species such as lipid oxidation products generated by reactive oxygen species to incorporation of BHZ was judged non-significant by comparing the fluorescence intensities in the absence and presence of vitamin E (50 μM). On the basis of probe’s proven validity, its suitability for detecting lysyl oxidase activity in pathological specimens was determined. For this purpose, aortic tissue was prepared from wildtype and heterozygous LOXL2 +/− mice of each young (< 3 months) and old (> 18 months) age. Tissue samples were incubated with BHZ and co-stained for LOXL2. Incorporation of BHZ was highest for aortic rings from old wild-type and lowest for young LOXL2 +/− mice, which correlates well with the LOXL2 expressions levels. Significant differences in the BHZ-derived fluorescence signal were also found between young and old mice each within the in the wild-type and LOXL2 +/− group.^[144]^

Biotin as affinity handle was also employed in the LOXL2-directed inhibitor-based probe **23**.^[145]^ Because of its 3-fluoroallylamine partial structure it is capable of irreversible covalent targeting and represents therefore an activity-based probe. The warhead is connected via a sulfone group to the aromatic core to which two biotin derived moieties were attached via a 3,4-dihydroxyphenylacetylene-derived linker each via Cu-catalysed alkyne-azide cycloaddition, while details on the synthesis of the compound were not published. Probe **23** was designed with the aim of proving target engagement of LOXL2 by detecting unoccupied enzyme with the probe by an ELISA-like method. Occupancy of LOXL2 in human plasma by a fluoroallylamine-based inhibitor related to compound **13** (bearing isopropyl in place of the methyl group and a 3-(6-(methylsulfonyl)pyridin-3-yl)methyl residue instead of the sulfonamide-functionalised phenyl ring) was probed after administration of each a single oral dose of 100, 200 or 400 mg of inhibitor. This has revealed that inhibited LOXL2 peaked at 4 h after gavage each in a dose-dependent extent, with an inhibition level of 80% reached for 100 mg, which dropped to 20% after 24 h due to resynthesis of the protein. The time profile of target occupancy of LOXL2 released into the blood resembled that in central organs as established in rat studies.^[145]^ Thus, the use of **23** for pharmacokinetic studies aptly illustrates the utility of activity-based probes for the translation of enzyme inhibitors into therapeutic application. Attempts towards employing **23** for microscopic imaging of LOXL2 in cell and tissue samples were not reported.

Activity-based detection of lysyl oxidases was also demonstrated for the alkyl hydrazine derivative **24**, which is equipped with a terminal 2,4-dinitrophenyl residue as affinity handle.^[146]^ The latter moiety can be visualised with a commercially available monoclonal antibody in a Western blot-like setting. By applying this methodology, the activity-based probe **24** enabled the detection of 150 ng of isolated LOX. Probe binding was attenuated by heptylhydrazine and caproic acid hydrazide in a dose-dependent manner^[146]^ that reflected their LOX-inhibitory activities determined in a fluorimetric activity assay.^[125b]^ The hydrazine warhead of **24** reacts with the LTQ cofactor in a substrate-analogous mode. The thereby initially formed quinohydrazone tautomerises to the corresponding stable azo compound,^[10,33]^ which accounts for irreversible probe binding. Differentiation between TPQ-dependent amine oxidases, which are also targeted by **24**, is ensured by electrophoretic separation of the enzyme-probe complex.^[146]^

### 5.2. Probes for MR-based imaging

**Figure 9.**
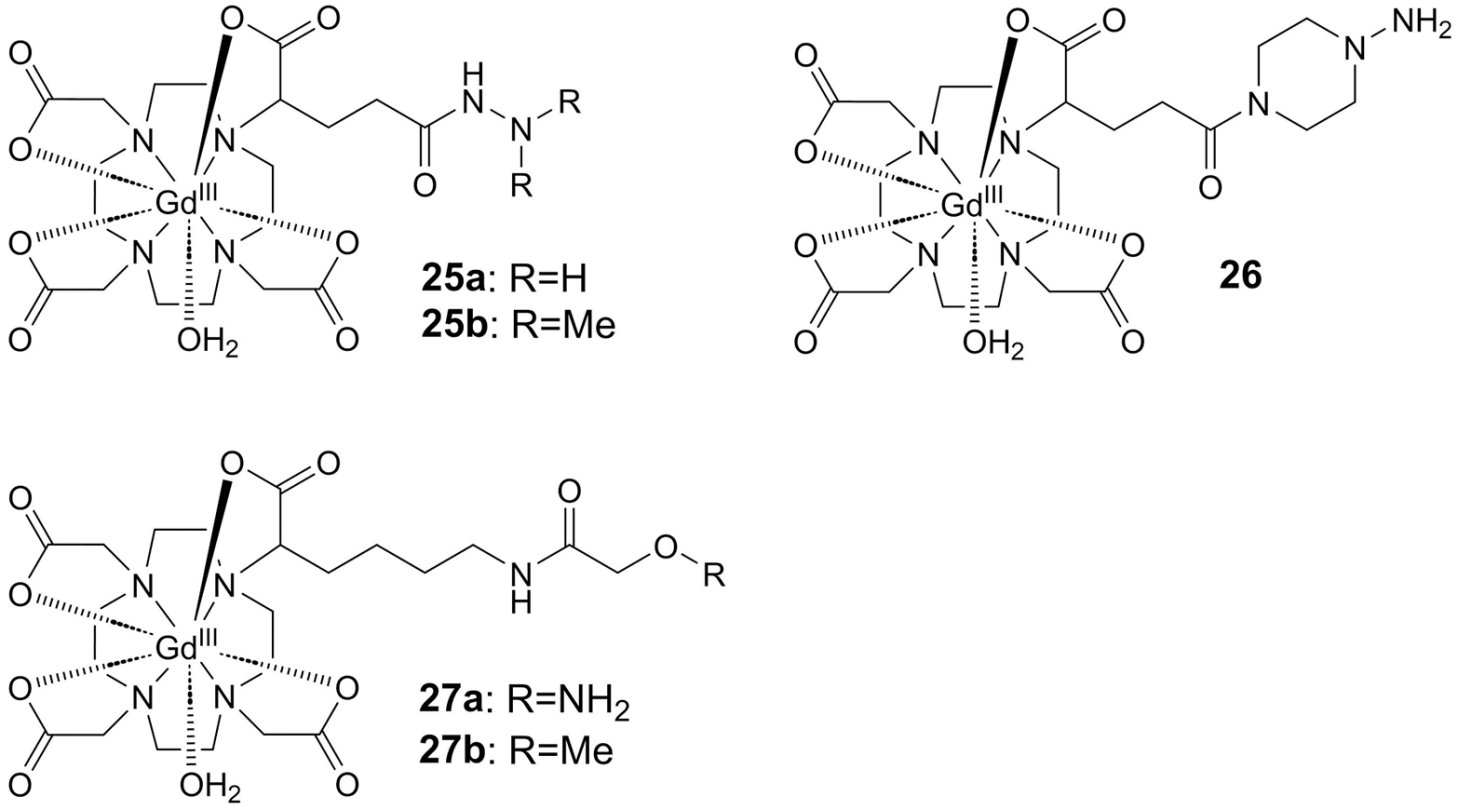
Probes for MR imaging of lysyl oxidase-generated allysine residues.

Probe molecules detecting the allysine-derived products of lysyl oxidase catalysis were also used for imaging in living organisms. The molecular imaging modalities that enable translation into clinical application are mainly magnetic resonance imaging (MRI), single-photon emission computed tomography (SPECT) and positron emission tomography (PET),^[147]^ which can be combined in hybrid systems such as PET/MRI or SPECT/MRI. MRI is based on the detection of tissue water protons by magnetic resonance and the image contrast arises from the different water content in various organs and tissue. Additional contrast on the basis of biochemical processes can be generated by bifunctional probe molecules which consist of an agent that shortens the relaxation times of the water protons, typically gadolinium ions, in addition to a moiety targeting biomacromolecules such as the protein of interest. The paramagnetic Gd^3+^ ion, which contains seven unpaired electrons, creates a strong local magnetic field fluctuating at the rate at which the probe molecule rotates in solution. The closer its rotation frequency is to the Larmor frequency of the surrounding water protons, the more efficiently their relaxation is enhanced, which in turn results in amplification of the MR signal.^[148]^ In order to design MRI probes capable of reacting with protein-bound allysine, 2,2’,2”,2’”-(1,4,7,10-tetraazacyclododecane-1,4,7,10-tetrayl)tetra-acetic acid (DOTA)-derived Gd^3+^-chelating moieties (DOTA-GA; GA = glutaric acid) were functionalised with hydrazine and hydroxylamine groups. The DOTA moiety functions as chelator for Gd^3+^ ions, which enhance the relaxivity of protons in the magnetic field, resulting in signal amplification. Further enhancement of relaxivity is caused by protein binding, which slows the rotational motion of the probe molecule. Regarding the hydrazine-derived agents of this type, hydrazide **25a** (Figure 9), in which hydrazine is directly attached to the DOTA-GA side chain, was initially reported.^[149]^ Increase in relaxivity resulting from protein binding of the probe by hydrazone formation was demonstrated for oxidised BSA, which has revealed a 3.4-fold increase compared to unbound **25a** in the absence of protein. Binding of the probe to porcine aortic tissue, for which an allysine content of 7.5 μmol/g was determined, occurred *in vitro* with a dissociation constant (*K*_d_) of 650 ± 61 μM. In contrast, the *N*,*N*-dimethylated analogue **25b** did not bind to the cultured tissue. On the basis of these results, **25a** was investigated in a mouse model of bleomycin-induced pulmonary fibrosis in comparison to its negative-control compound **25b**. Application of **25a** and **25b** to bleomycin-injured mice has revealed a change in contrast-to-noise ratio (ΔCNR) for the lung in comparison to skeletal muscle tissue that was 3.8-fold higher for **25a** in relation to **25b**. A similar ΔCNR ratio was obtained when the **25a**-enhanced MR images of bleomycin- and sham-treated mice were compared. Treatment of the injured mice with βAPN attenuated the ΔCNR level to the range of the sham-treated mice. Furthermore, **25a**-enhanced MR imaging was demonstrated to be suitable for monitoring progression of the fibrotic disease, since the measured ΔCNR for different treatment times with bleomycin correlated well with the allysine concentration, expression of LOXL2 and total lysyl oxidase activity in the lungs of the treated mice. Similar results were obtained when **25a** was administered in the mouse model of CCl_4_-induced hepatic fibrosis. Notably, owing to the fast renal clearance (blood half-life in vivo: 4.7 min^[150]^) no significant liver enhancement was observed for the vehicle-treated animals, while ΔCNR was 24-fold higher after treatment with CCl_4_ for 12 weeks.^[149]^

Based on the short in vivo half-life of **25a** due to rapid clearance, the authors concluded that increasing the reactivity of the hydrazine moiety could increase signal enhancement in the MR images. For this purpose, *N*-amino piperazine derivative **26** (Figure 9), which bears a more nucleophilic hydrazine group, was designed and synthesised.^[150]^ The increased reactivity was confirmed on the basis of its reaction with 2-formylpyridine as model aldehyde, for which a second-order rate constant (*k*_on_) of 0.52 M^−1^s^−1^ was determined, which is 11 times higher than the *k*_on_ value for **25a** in the reaction with this aldehyde. The higher rate contributes to a decreased dissociation constant of 45 μM for the hydrolysis of the **26**-derived hydrazone, which accounts for a 9 times higher thermodynamic stability compared to the corresponding product derived from hydrazide **25a**. Applying the murine model of bleomycin-induced lung fibrosis, compound **26** was biologically characterised in a similar manner as **25a**. This has revealed that the Gd complex **26** led to a 12-fold higher ΔCNR for bleomycin-treated mice versus naïve animals compared to a ratio of 3.7 for 25a. These results indicate that the higher nucleophilicity of hydrazine derivative **26** results in a significantly more favourable in vivo performance.^[150]^

**Figure 10.**
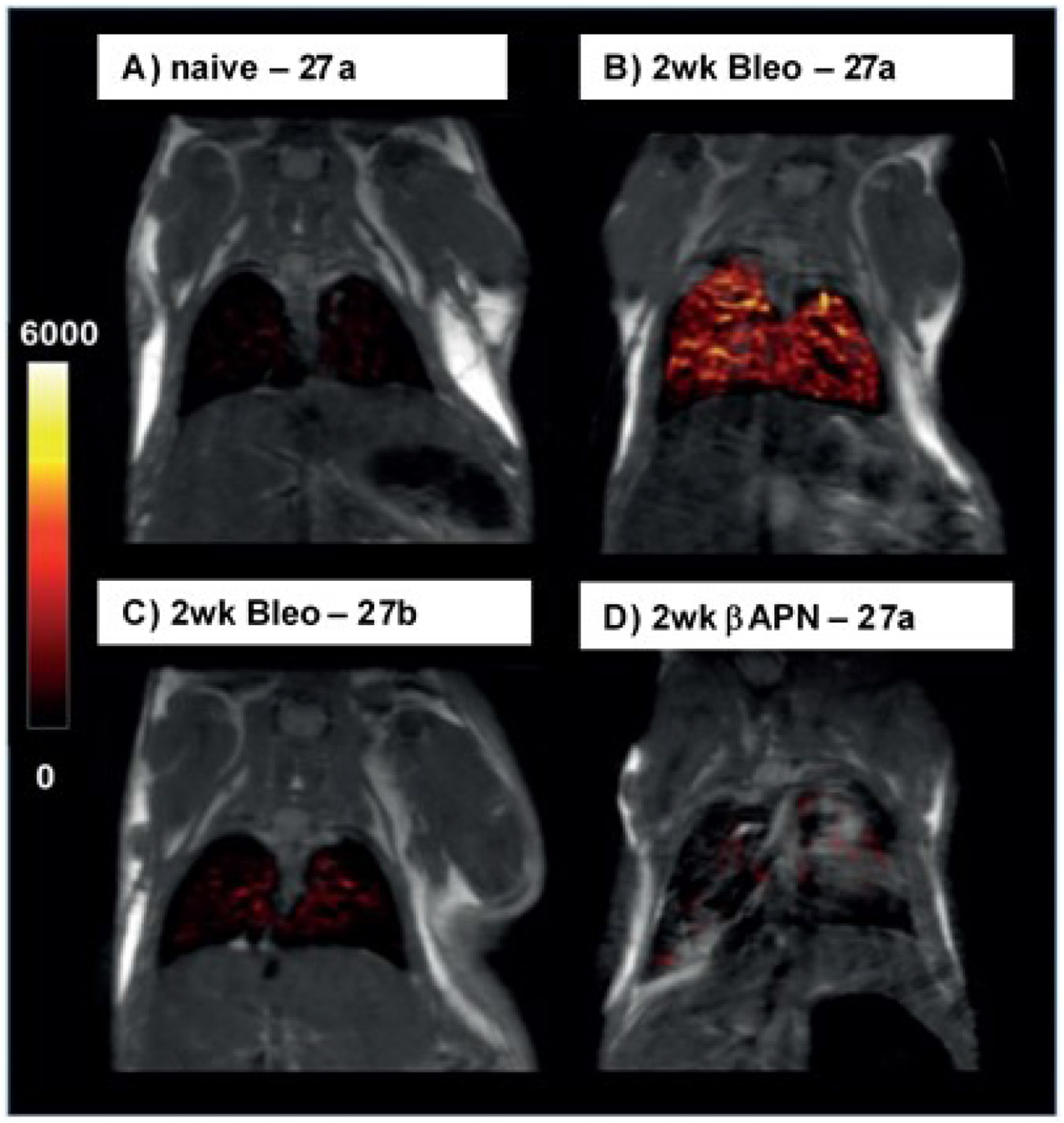
Reprinted MRT images showing the uptake of aminoxyfunctionalised Gd complex **27a** and analogous unreactive methyl ether **27b** as negative-control probe in the murine model of bleomycin-induced fibrosis. A) Uptake of **27a** in naive mouse, showing low MR signal enhancement in healthy lungs; B) GdOA uptake in bleomycin-treated mouse, showing strong lung enhancement in 14 day bleomycin-injured mice; C) uptake of **27b** in bleomycin-challenged mouse, showing low lung enhancement in 14 day bleomycin-injured mice with negative control probe and D) uptake of **27a** in bleomycin-challenged mouse dosed daily for 14 days with βAPN, showing little lung enhancement, indicating an absence of allysine. Reproduced (Figure 2 in Ref. [151]) and edited (selection of subfigures, subfigure titles, compound reference numbers, caption text) from Waghorn *et al.*^[151]^ with permission, Copyright © 2017, Wiley-VCH Verlag GmbH & Co. KGaA, Weinheim (Germany).

Changing the hydrazine functionality to *O*-alkylhydroxylamine, as realised in Gd complex **27a** (Figure 9), leads to improved allysine reactivity compared to **25a**, as indicated by a higher affinity to allysine-rich porcine aorta (*K*_d_ 360 μM vs 650 μM).^[151]^ The ratio of probe accumulation in the lung tissue of naïve and bleomycin-treated mice was determined each for **27a** and **26** by assaying the Gd content using ICP-MS. This revealed ratios of 2.4 and 14, respectively, which suggests the superiority of **26** as allysine-reactive in vivo probe. No difference in the change in lung/muscle ratio for the MR signal was obvious between the naïve and bleomycin-treated mice administered with probe **27a** and its analogous methyl ether **27b** (bearing a methyl group in place of the primary amino group, Figure 9), respectively, which indicates the specificity of **27a** *in vivo.*^[151]^ The favourable performance of **27a** is impressively reflected in the reprinted small animal MRT images shown in Figure 10.

### 5.3. Probes for PET imaging (radiotracers)

#### 5.3.1. ^68^Ga-Labelled allysine-reactive gallium complexes

**Figure 11.**
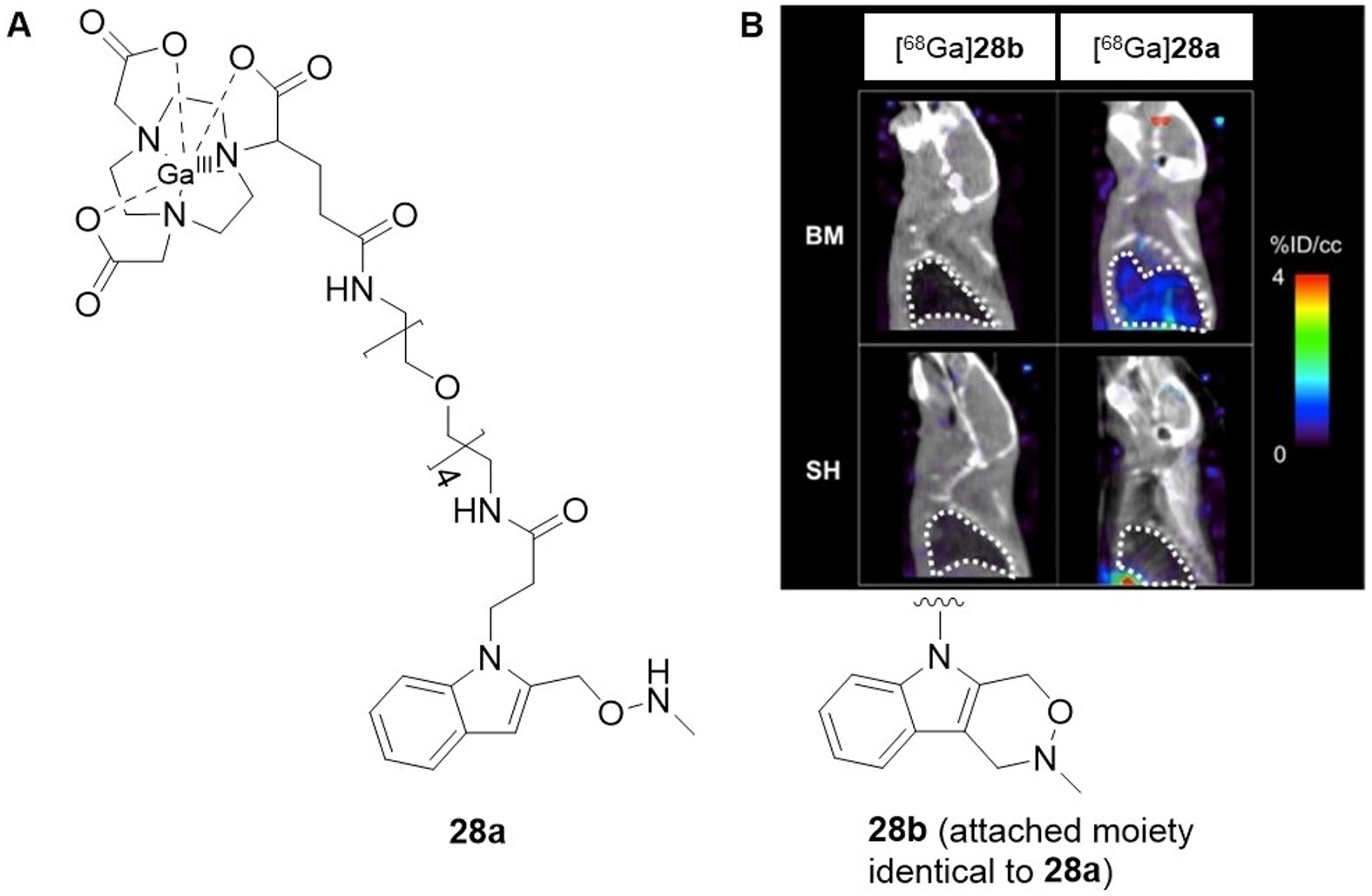
A) Chemical structures of probe **28a** and control compound **28b** for PET imaging of lysyl oxidase-generated allysine residues. B) Reprinted representative fused PET-CT (colour and gray scale, respectively) sagittal images of the head and thorax 110–120 min p.i. of (left) [^68^Ga]**28b** and (right) [^68^Ga]**28a** in sham- (SH, bottom) and bleomycin-injured (BM, top) animals; the lung is highlighted with a dashed white line. The PET signal expressed as %ID/cc, in the lung is low in both sham and BM injured mice with [^68^Ga]**28b**, but is higher in the BM injured mouse imaged with [^68^Ga]**28a** demonstrating the specificity of the latter probe for fibrogenesis. Reproduced (Figure 3 in Ref. [155]) and edited (compound reference numbers, caption text) from Wahsner *et al.* with permission, Copyright © 2019, American Chemical Society.

The Caravan group also employed aldehyde-reactive probes for developing allysine-detecting PET tracers. Unlike MRI-based imaging probes, which are administered in doses of 0.1-0.3 mmol kg^−1^ and should be completely eliminated from the body after image acquisition in order to prevent the accumulation of potentially toxic Gd^3+^,^[152]^ probes labelled with radionuclides of short half-life such as used for PET imaging do not cause harm in this respect because they are usually applied at much lower doses in the range of 1–1000 pmol kg^−1^.^[153]^ Therefore, a probe that allows an irreversible reaction with the aldehyde can be applied instead of reversible hydrazone and oxime formation. For this purpose, a Pictet-Spengler type reaction was chosen,^[154]^ that is enabled by the 2-methylaminoxyindole derivative **27a** (Figure 11).^[155]^ This probe can undergo a cyclocondensation reaction with aldehydes under the formation of stable C─N and C─C bonds. For butyraldehyde as model compound this reaction proceeds with second-order rate constant of 0.28 M^−1^s^−1^. Metalation of the 1,4,7-triazacyclononane,1-glutaric acid-4,7-acetic acid (NODAGA) chelator moiety was performed with both ^nat^Ga^3+^ and the positron-emitting radionuclide gallium-68 ([^68^Ga]Ga^3+^, T_1/2_ = 68 min). Biological evaluation of **28a** was performed in a similar way to the MRI probes. Binding of [^nat^Ga]**28a** to allysine-rich porcine aorta was quantified on the basis of ICP-MS, which allowed for determining a binding range of 2–2.5 nmol/mg, while binding under pretreatment with the unconjugated probe (propionic acid derivative lacking linker and chelator) was about one order of magnitude lower. A similar degree of binding was determined for the unreactive analogue [^nat^Ga]**28b** (formaldehyde-derived Pictet-Spengler product). Performing HPLC-ICP-MS analysis, both [^nat^Ga]**28a** and [^nat^Ga]**28b** were proven to be entirely stable in human blood plasma for 24 h at 37°C. Performing PET-imaging in the murine model of bleomycin-induced pulmonary fibrosis has revealed that the uptake of [^68^Ga]**28a** at 120 min p.i. in the injured lung is 7-fold higher than its uptake in the lung tissue of sham-treated animals. A similar ratio was obtained when the radiotracer uptakes of [^68^Ga]**28a** and [^68^Ga]**28b** were compared. Notably, a linear correlation between the lung uptake of [^68^Ga]**28a** and the allysine content of the lung tissue could be derived. The basic pharmacokinetic properties were similar for both probes, as indicated by similar halflives for blood clearance and shared elimination pathways (T_1/2_ = 6-9 min), which are predominantly renal excretion and partly hepatobiliary elimination.^[155]^

#### 5.3.2. ^18^F-labelled telopeptide derivatives as substrate-based radiotracers

**Figure 12.**
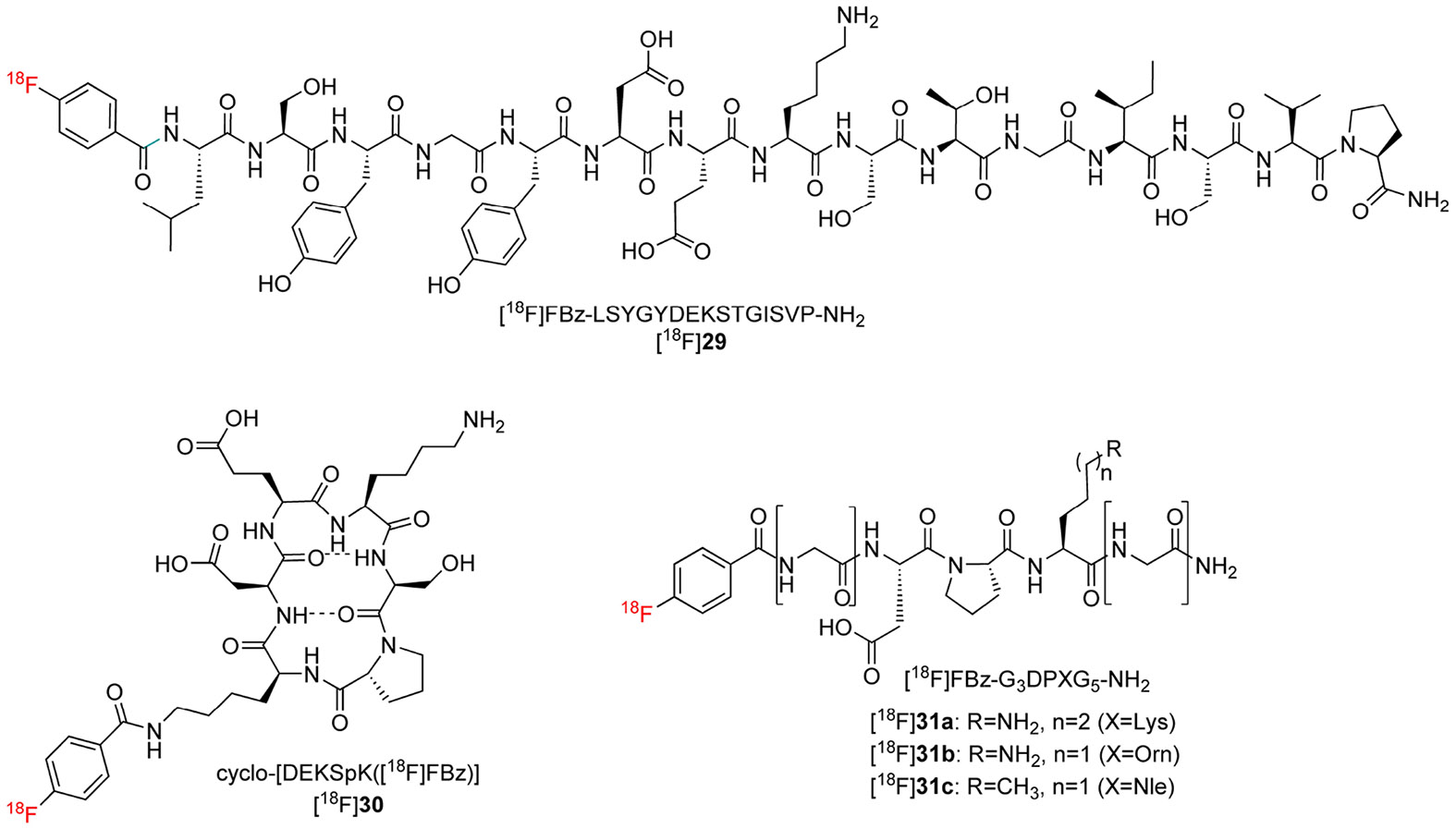
Substrate-based radiotracers for PET imaging of lysyl oxidases

**Figure 13.**
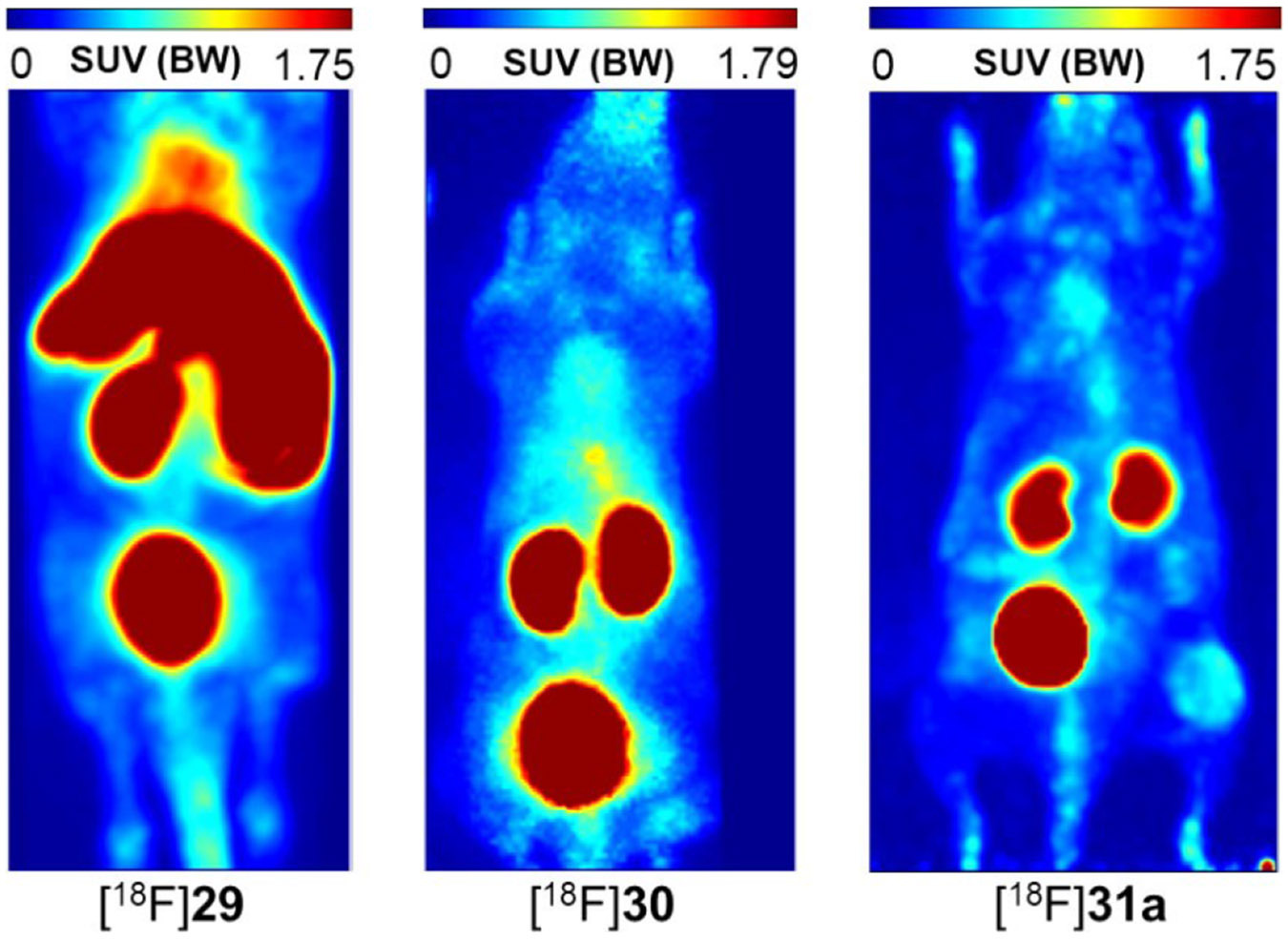
Small animal PET images (maximum intensity projections) of A375 tumour-bearing NMRI (nu/nu) mice showing the distribution of [^18^F]**29**, [^18^F]**30** and [^18^F]**31a** each after 20 min p.i. The location of the tumour tissue is indicated by an arrow. Reproduced (Figure 7 in Ref. [156]) and edited (arrangement of panels, compound reference numbers, caption text) from Kuchar *et al.*^[156]^ with permission, Copyright © 2018, Kuchar *et al.*

Regarding PET imaging of lysyl oxidase activity using agents that directly target the enzyme, radiotracers were designed on the basis of compounds derived from the N-terminal telopeptides of the α1 chain of type I collagen.^[156]^ This approach was initially based on the working hypothesis that these tracer compounds would undergo lysyl oxidase-mediated cross-linking with endogenous collagen strands. Besides the authentic α1(I) N-telopeptide, whose N-terminal pyroglutamate residue was removed to enable labelling with fluorine-18 (T_1/2_ = 109.8 min) by N-terminal ^18^F-fluorobenzoylation, a linear undecapeptide with favourable substrate properties^[36]^ and a derived cyclohexapeptide were synthesised (Figure 12). The latter was shown to exhibit the central α1(I) N-telopeptide DEKS motif in a β1 turn,^[157]^ which matches the conformation of the telopeptide chain in the complex with its receptor region of the triple-helical type I collagen.^[158]^ Site-selective radiolabelling was achieved by reacting the corresponding resin-attached peptidic precursors containing the free terminal amino group and otherwise completely protected functional groups with the prosthetic labelling agent *N*-succinimidyl 4-[^18^F]fluorobenzoate ([^18^F]SFB). This was followed by one-step side-chain deprotection and release of the ^18^F-labelled peptides from the polymeric support.^[159]^ With regard to the potential cross-linking of the substrate-based radiotracers *in vivo*, the interaction of nonradioactive telopeptide derivatives with type I atelocollagen was investigated by surface plasmon resonance (SPR). These experiments have revealed that the telopeptide-collagen interaction is of a very weak character and a lysyl oxidase-mediated cross-linking of the ^18^F-labelled substrate peptides with endogenous collagen is very unlikely. Studies on the ex vivo biodistribution and stability of the three ^18^F-fluorobenzoylated peptides in normal Wistar rats have shown that there is no irreversible accumulation in organs and the radiotracers are predominantly excreted renally. The ^18^F-labelled telopeptide [^18^F]**29** is subject to relatively rapid degradation in vivo, the DPK peptide [^18^F]**31a** can be considered sufficiently stable and for the cyclic analogue [^18^F]**30** no degradation products were detected over a period of 60 min. The expression of the lysyl oxidase isoforms LOX and LOXL2 in human A375 melanoma cells were confirmed both at the level of the isolated cells and in the tumour tissue by Western blot analysis, demonstrating the suitability of the corresponding xenograft model. Radiopharmacological investigation in this model was performed by dynamic PET studies, ex vivo radioluminography and biodistribution studies. Uptake in A375 tumour tissue was greatest for the DPK peptide [^18^F]**31a** among the three ^18^F-labelled telopeptide analogues, with maximum SUV levels reached after 10 min according to time-activity curves derived from PET measurements. The superior in vivo performance of [^18^F]**31a** among the ^18^F-labelled telopeptide analogues becomes visible by comparing their PET images 20 min p.i. (Figure 13). Irreversible accumulation in tumour tissue does not occur, consistent with the transient nature of the enzyme-substrate interaction. Treatment of A375 tumour-bearing mice with the lysyl oxidase inhibitor βAPN did not result in a reduction of radiotracer uptake in the tumour. To demonstrate that uptake of [^18^F]**31a** in A375 tumours is mediated by lysyl oxidases, analogues of this radiotracer were synthesised and ^18^F-labelled with the lysine residue replaced by ornithine ([^18^F]**31b**) and norleucine ([^18^F]**31c**, Figure 12). Tumour uptake of all three peptides was compared using ex vivo biodistribution in the A375 xenograft mice. This showed that the uptake of [^18^F]**31b** and [^18^F]**31c** in tumour tissue is significantly lower than that of [^18^F]**31a** and varies according to their substrate properties towards lysyl oxidases.^[156]^

As outlined above, by far the most findings with regard to the function of lysyl oxidase in tumour progression were obtained in the context of breast cancer. For this reason, [^18^F]**31a**, which was the best-performing compound in the melanoma model, was selected for evaluation in xenograft- and allograft-based murine breast cancer-models.^[116]^ Specifically, the xenograft models in NIH III nu/nu mice derived from the human cell lines MDA-MB-231 and MCF-7, and a syngeneic tumour allograft model in BALB/c mice based on the murine cell line EMT-6 were chosen for dynamic PET investigations with [^18^F]**31a**.

**Figure 14.**
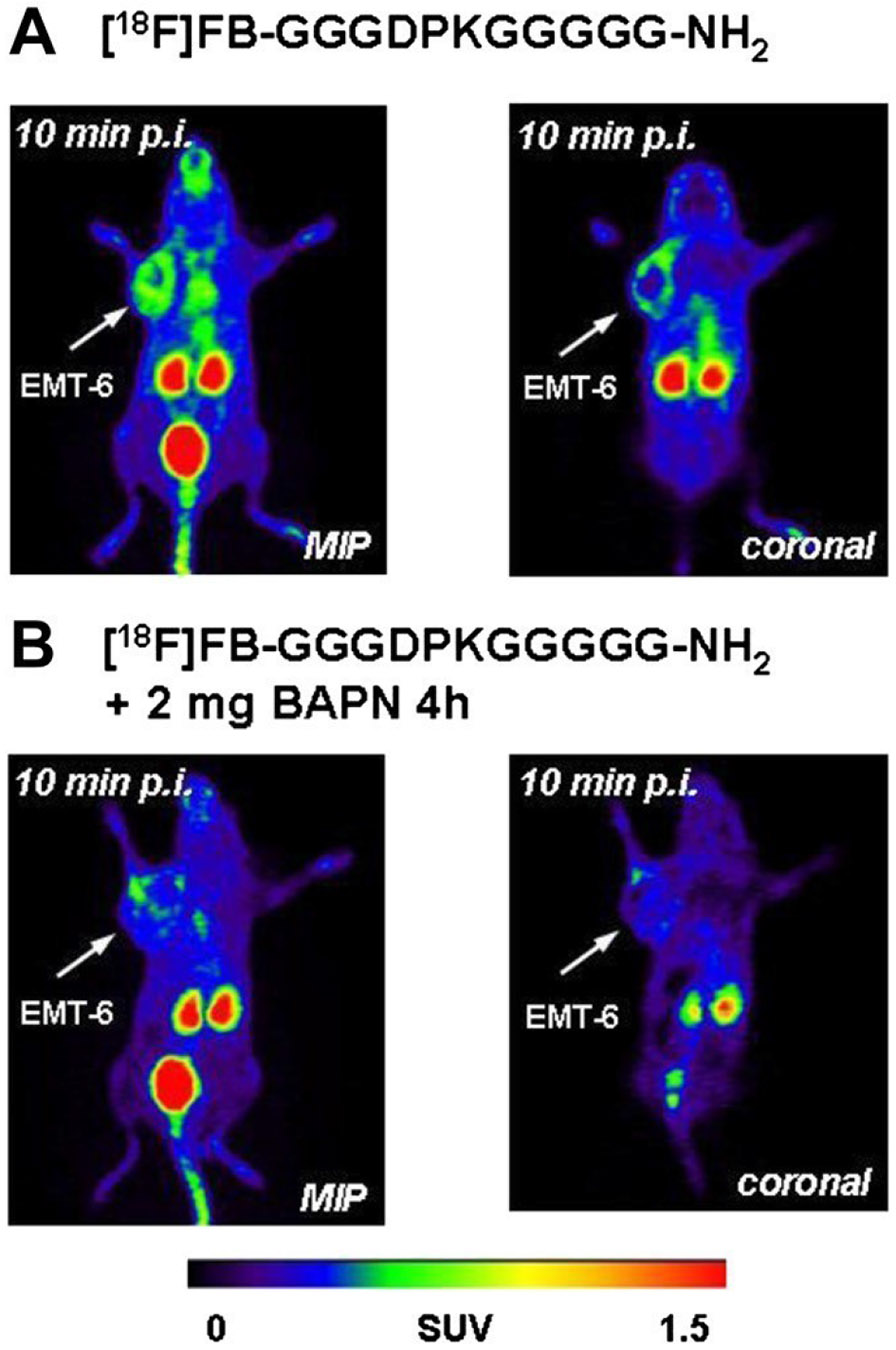
Reprinted representative PET images after injection of [^18^F]**31a** in the absence (A) and presence (B) of β-aminopropionitrile (βAPN) in EMT-6 tumour-bearing mice at 10 min p.i. Images are shown as maximum intensity projections (MIPs) and coronal slices from the tumour region. Reproduced (Figure 10 in Ref. [116]) and edited (selection of content, caption text) from Wuest *et al.*^[116]^ with permission, Copyright © 2015, Wuest *et al.*

The shape of the time-activity curves derived from the measurements for the tumour region was similar to the curve obtained in the melanoma model for all three breast cancer models. The maximum SUV values obtained were somewhat lower and the maxima appear slightly shifted to earlier time points around 5 min p.i. compared to the activity profile observed in the A375 tissue (Figure 9, bottom, in^[116]^). The highest uptake was observed for the syngeneic EMT-6 model with an SUV value of 0.63. The activity level in the muscle tissue was consistently lower, with the most favourable tumour-to-muscle ratio being achieved for the EMT-6 model with a value of 2.7 after 5 min. Even though the tracer accumulation in the tumour tissue was rather low, similar to the melanoma model, it was nevertheless reflected in the small animal PET images. The greatest image contrast for the tumour region emerged for the murine EMT-6 tumours, which is in accordance with the time-activity curves. Treatment of the EMT-6-bearing animals with βAPN at a dose of 100 mg/kg 4 h before injection of [^18^F]**31a** resulted in significantly reduced tumour uptake. In particular, the SUV value 5 min p.i. was 67% compared to that in the absence of βAPN. The attenuating effect of inhibitor administration on the uptake of [^18^F]**31a** is also visible when the PET images after 10 min p.i. are compared (Figure 14). Hence, the results of the radiopharmacological investigations of the radiolabelled DPK peptide [^18^F]**31a** in the three different murine breast cancer models suggest that this radiotracer is subject to tumour accumulation which is partially mediated by lysyl oxidases, to a similar extent as in the A375-derived melanoma model.^[116]^ This conclusion encourages the design of radiotracers based on irreversible inhibitors derived from compound **31a** in order to enhance target rentention and, consequently, image contrast of the probe.

#### 5.3.3. Application of other radiolabelled compounds in lysyl oxidase research

Apart from the radionuclide-based probes for molecular imaging referred herein, the application of radiotracer-based methods has contributed significantly to the discovery and study of lysyl oxidases. The addition of l-[U-^14^C]lysine to the culture medium with which aortas prepared from chicken embryos were incubated enables the generation of ^14^C-labelled elastin. This could be converted to ^14^C-allysine-containing elastin using embryonic chicken bone homogenate as the enzyme source. From this material, ^14^C-labelled aminoadipic acid was isolated after subjecting the protein to acidic hydrolysis and oxidation with performic acid.^[2]^ The quantity of this radiolabelled degradation product was considerably lower in the presence of βAPN. The corresponding use of d,l-[6-^3^H_2_]lysine as a radiotracer leads to the release of [^3^H]H_2_O during the catalytic cycle of lysyl oxidase without requiring protein hydrolysis, which was separated by distillation and quantified radiometrically as a surrogate for the enzyme activity. Accordingly, the presence of βAPN led to a drastic reduction in the detectable tritium activity.^[2]^ The use of the isotopomeric d,l-[4,5-^3^H_2_]lysine also leads to the release of tritium in the form of [^3^H]H_2_O by isotope exchange due to the C─H acidic position in the protein-bound allysine formed. Since this radiotracer is available in higher molar activity than d,l-[6-^3^H_2_]lysine, its application results in higher sensitivity.^[7a]^

Noteworthy, radiotracer methods in the context of lysyl oxidases were not only used for detecting their enzymatic activity. The ^14^C-labelling of LOX by reductive *N*,*N*-dimethylation on lysyine residues with [^14^C]formaldehyde made it possible to detect the translocation of extracellular LOX into the nucleus of vascular smooth muscle cells. This trafficking behaviour of LOX was mentioned in chapter 2 (see above).^[47]^

## 6. Summary and Outlook

The properties of lysyl oxidases were summarised from a chemical and structural point of view. The biochemical basis for their function as key enzymes of tumour progression and metastasis was highlighted. Recent development of subtypespecific inhibitors should enable clinical trials towards lysyl oxidase inhibition for treatment of neoplastic and fibrotic diseases. Recently discovered imaging probes targeting lysyl oxidases potentially allow for the diagnostic characterisation of malign tumours and facilitate the clinical translation of inhibitors. Furthermore, the clinical diagnosis of cancer and fibrotic diseases will be improved by allysine-reactive probes and lysyl oxidase-directed tracers.

## Figures and Tables

**Table 1.** Sequence identity and homology of human lysyl oxidase isozymes (reproduced as published Amendola *et al.*^[30]^).

	LOX	LOXL1	LOXL2	LOXL3	LOXL4
LOX	100	77/78	49/68	52/67	52/67
LOXL1		100	49/66	54/65	51/64
LOXL2			100	72/88	71/86
LOXL3				100	72/87
LOXL4					100

**Table 2.** Compilation of selected literature reports on the involvement on the expression and functions of the lysyl oxidase isozymes LOX and LOXL2 in selected tumour entities of various origins.

Tumour entity	isoform	reference
Breast cancer	LOX	[53]
	LOXL2	[50c, 54]
Lung cancer	LOX	[55]
	LOXL2	[56]
Colorectal carcinoma	LOX	[57]
	LOXL2	[58]
Pancreatic carcinoma	LOX	[59]
	LOXL2	[59a, 60]
Gastric cancer	LOX	[51b]
	LOXL2	[61]
Prostate cancer	LOX	[62]
	LOXL2	[63]
Glioma	LOX	[64]
	LOXL2	[65]
Head /neck tumours (squamous cell type)	LOX	[66]
	LOXL2	[67]
Hepatocellular carcinoma	LOX	[68]
	LOXL2	[69]
Skin cancer (w/o melanoma)	LOX	[70]
	LOXL2	[56a]

## Data Availability

Data sharing is not applicable to this article as no new data were created or analyzed in this study.
